# Indoor Emission, Oxidation, and New Particle Formation of Personal Care Product Related Volatile Organic Compounds

**DOI:** 10.1021/acs.estlett.4c00353

**Published:** 2024-08-30

**Authors:** Tianren Wu, Tatjana Müller, Nijing Wang, Joseph Byron, Sarka Langer, Jonathan Williams, Dusan Licina

**Affiliations:** † Human-Oriented Built Environment Lab, School of Architecture, Civil and Environmental Engineering, École Polytechnique Fédérale de Lausanne, CH-1015 Lausanne, Switzerland; ‡ Civil and Architectural Engineering and Construction Management, University of Cincinnati, Cincinnati, Ohio 45221, United States; § Atmospheric Chemistry Department, Max Planck Institute for Chemistry, Hahn-Meitner-Weg 1, 55128 Mainz, Germany; ∥ IVL Swedish Environmental Research Institute, Environmental Chemistry, SE-400 14 Göteborg, Sweden; ⊥ Chalmers University of Technology, Department of Architecture and Civil Engineering, Division of Building Services Engineering, SE-412 96 Göteborg, Sweden

**Keywords:** Indoor ultrafine particles, Oxidized vapors, Ozone, Inhalation exposure, Chamber study

## Abstract

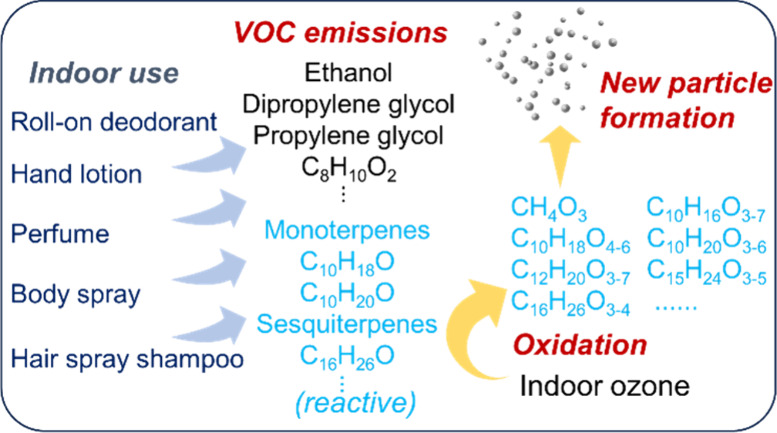

Personal care products (PCPs) contain diverse volatile organic compounds (VOCs) and routine use of PCPs indoors has important implications for indoor air quality and human chemical exposures. This chamber study deployed aerosol instrumentation and two online mass spectrometers to quantify VOC emissions from the indoor use of five fragranced PCPs and examined the formation of gas-phase oxidation products and particles upon ozone-initiated oxidation of reactive VOCs. The tested PCPs include a perfume, a roll-on deodorant, a body spray, a hair spray, and a hand lotion. Indoor use of these PCPs emitted over 200 VOCs and resulted in indoor VOC mixing ratios of several parts per million. The VOC emission factors for the PCPs varied from 2 to 964 mg g^–1^. We identified strong emissions of terpenes and their derivatives, which are likely used as fragrant additives in the PCPs. When using the PCPs in the presence of indoor ozone, these reactive VOCs underwent oxidation reactions to form a variety of gas-phase oxidized vapors and led to rapid new particle formation (NPF) events with particle growth rates up to ten times higher than outdoor atmospheric NPF events. The resulting ultrafine particle concentrations reach ∼34000 to ∼200000 cm^–3^ during the NPF events.

## 1 Introduction

Personal care products (PCPs) encompass consumer products used for personal hygiene, grooming, and beautification and are widely used indoors. These products exhibit a wide variety of chemical compositions. Headspace analyses have demonstrated that PCPs emit hundreds of volatile organic compounds (VOCs).^1−3^ Notable VOCs commonly observed include monoterpenes, acetaldehyde, siloxanes, alcohols (e.g., ethanol, n-propanol), and alkanes (e.g., butane).^4−6^ Indoor use of PCPs is therefore a potentially important source of human exposure to VOCs. The use of PCPs can result in episodic strong emission events, causing indoor VOC levels to reach one or 2 orders of magnitude higher than those found outdoors.^7^

Monoterpenes (C_10_H_16_) are a class of VOCs commonly added to personal care products (PCPs) as fragrances,^8^ that are highly reactive with gas-phase oxidants.^9−12^ The reactions between monoterpenes and ozone play an important role in indoor air chemistry, as they represent a substantial gas-phase loss of ozone, induce the formation of secondary VOCs, and initiate new particle formation. The use of products like air fresheners, diffusive oils, and cleaning products can release monoterpenes, such as lemon-scented limonene, which subsequently react with indoor ozone, leading to the formation of secondary organic aerosols (SOAs) within the building and increasing the occupants’ inhalation exposure to particles.^13−15^ Using fragranced PCPs indoors may also lead to particle formation in a similar manner. Moreover, the ozonolysis of monoterpenes leads to the production of reactive oxygen species (ROSs), such as organic peroxides and hydroperoxides, which can exist in both gas and particle phases.^16^ Due to their biochemical reactivity, ROSs can damage lung cells, potentially resulting in adverse health effects for individuals exposed to these compounds.^17,18^ On the other hand, indoor emitted VOCs from PCPs may be transported outdoors due to building ventilation and contribute to atmospheric organic gases and the formation of SOAs, which has been addressed in several recent modeling and chamber studies.^19−32^

A better understanding is needed regarding indoor emissions of VOCs from the use of PCPs and the subsequent formation of oxidized organic vapors and particles due to secondary chemistry with ozone. The objective of this study is to provide novel insights into how using PCPs indoors alters the chemical composition of indoor air through a series of chamber experiments, where we used online high-resolution mass spectrometers and cutting-edge aerosol instrumentation to characterize the VOC emission dynamics, transient human inhalation exposure, formation of gas-phase oxidation products upon ozone-initiated oxidation, and new particle formation from the use of selected fragranced PCPs indoors.

## 2 Methods and Materials

### 2.1 Chamber and Experimental Description

The experiments were conducted in the indoor environmental chamber at EPFL Fribourg, Switzerland. The chamber represents a modern office environment with an area of 24.7 m^2^ and a volume of 62 m^3^. The chamber adopts a single-pass mechanical ventilation system, providing 100% outdoor air. It is equipped with two particulate filters (F7 and high-efficiency particulate filters) and one activated carbon filter to remove particles, ozone, and VOCs in the supply air. The PCPs being tested were randomly selected at a grocery store, including a deodorant body spray (hereafter body spray), a hand lotion, a roll-on deodorant, a perfume, and a dry shampoo hair spray (hereafter hair spray). Details about the type of PCPs and their ingredient chart are presented in the Supporting Information (SI).

Two types of experiments were conducted. Primary emission experiments examined the direct VOC emissions resulting from the use of PCPs, where the ventilation system maintained the chamber air exchange rate (AER) at 3 h^–1^. The second type of experiments—oxidation experiments—probed the ozone-initiated oxidation of VOCs originating from the PCPs, where an ozone generator maintained the chamber’s ozone level at 35–40 ppb with an AER of 0.65 h^–1^. In the experiments, the application of the PCPs was simulated in the chamber by a volunteer, who wore a protective suit, activated carbon facemask, and nitrile gloves to minimize human-related VOC emissions and ozone-human surface reactions. For the body spray, perfume, and hair spray, the volunteer directly sprayed these products in the chamber air at a height of 160 cm above the floor. For the hand lotion and roll-on deodorant, the products were applied and spread on a Kimwipe (40 × 40 cm^2^) on a clean glass plate using a glass rod in the chamber. The glass plate with the Kimwipe was left in the chamber for an hour. In this study, the VOC emissions from the hand lotion and roll-on deodorant only represent the emissions within the first hour of application. The products were weighed on a balance to calculate the consumption before and after each use.

### 2.2 Measurements and Instrumentation

In the chamber, the aerosol size distribution over the size range of 1.4 nm to 10 μm was monitored in real-time with an A11 nanocondensation nucleus counter (nCNC; Airmodus Ltd., Helsinki, Finland; 1.4–3 nm), a scanning mobility particle sizer (SMPS; Grimm Aerosol Technik, Hamburg, Germany; 3–55 nm), and a wide-range aerosol spectrometer (MiniWRAS; Grimm Aerosol Technik, Hamburg, Germany; 55–10000 nm). Ozone and NO_*x*_ concentrations were monitored by a Tanabyte 72X analyzer (Tanabyte Engineering, Inc., FL, USA) and a 2B Tech 405 analyzer (2B Technologies, CO, USA), respectively.

A Vocus proton-transfer-reaction mass spectrometer (Aerodyne Research Inc., MA, USA; hereafter Vocus PTR) and a Vocus iodide-adduct chemical ionization mass spectrometer (Tofwerk AG, Thun, Switzerland; hereafter I-CIMS) with an Aim reactor were deployed to measure the gas-phase organic compounds. All the experiments were repeated with each instrument, except for the hand lotion and roll-on deodorant primary and oxidation experiments, which were only conducted with the Vocus PTR. We estimated the volume mixing ratio for the VOCs monitored by the Vocus PTR based on measured or assumed instrument sensitivities (SI). The abundance of the compounds monitored by the I-CIMS was reported as counts per second (cps). During the primary emission experiments, the chamber air was also sampled using Tenax TA sorbent cartridges (PerkinElmer) and analyzed with a Thermal-Desorption Gas-Chromatography Mass-Spectrometer (TD-GC-MS; Markes International, Unity 1 and Ultra thermal desorber; Agilent 6890 gas chromatograph; Agilent 5973 N mass selective detector). Details about sampling and the operation of the instruments are presented in the Supporting Information (SI).

## 3 Results and Discussion

### Primary Emissions of VOCs

**Figure 1:**
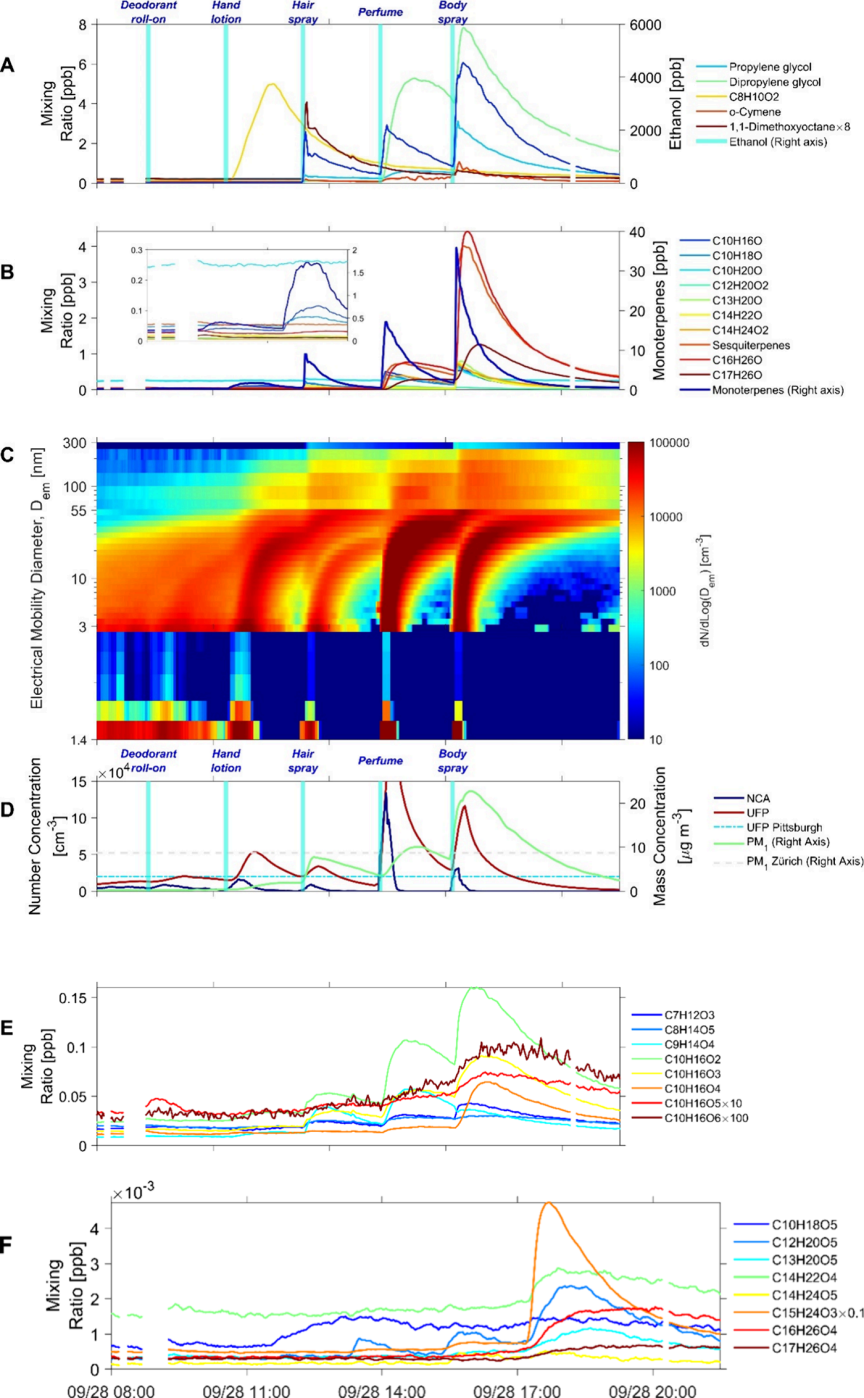
(A, B) Concentrations of directly emitted VOCs from the PCPs measured by the Vocus PTR; (C) Time-series plot of aerosol number size distribution (dN/dLog(D_em_)); (D) concentrations of nanocluster aerosol (NCA, <3 nm; blue line), ultrafine particle (UFP, 3–100 nm; red line), PM_1_ mass concentration (<1 μm; green line) with a reference UFP concentration measured in urban Pittsburgh^44^ (blue dash-dotted line) and a PM_1_ mass concentration measured in urban Zürich^45^ (gray dashed line); and (E–F) concentrations of selected gas-phase oxidation products measured by the Vocus PTR in an oxidation experiment. The consumption of the roll-on deodorant, hand lotion, hair spray, perfume, and body spray were 0.264, 9.72, 2.04, 0.167, and 1.11 g, respectively. The vertical bars in Panels A and D indicate when the PCPs were applied. The ozone level was between 35 and 40 ppb.

We were able to differentiate the directly emitted VOCs and secondarily formed gas-phase organic compounds by comparing the concentration profiles obtained during the primary emission and oxidation experiments. The Vocus PTR identified more than 200 directly emitted VOCs associated with the selected PCPs. Figure 1A illustrates the concentration profiles of several directly emitted VOCs with low indoor reactivities in an oxidation experiment. The molecular formulas in the legend were determined based on the mass-to-charge ratio measured by the Vocus PTR, while the compound names in the legend were assigned based on TD-GC-MS analysis (Table S1).

Among the “spray-type” PCPs, namely hair spray, perfume, and body spray, significant emissions of ethanol were observed. At an AER of 0.65 h^–1^, the ethanol concentration reached 2000–4000 ppb (2–4 ppm) immediately after applying the three PCPs. However, the concentrations were still several orders of magnitude lower than the occupational short-term exposure limit of 1000 ppm.^33^ Strong emissions of propylene glycol (PG) and dipropylene glycol (DPG) were detected from the perfume and body spray. These compounds are commonly used as solvents and carriers for fragrant chemicals in cosmetic products and PCPs.^34^ Ethanol facilitates rapid evaporation, providing an initial burst of fragrances, while PG and DPG serve as fixatives, prolonging the on-body evaporation.^35−37^ Additionally, 1,1-dimethoxyoctane^38^ and o-cymene were two fragrances emitted from the hair spray and body spray, respectively. The hand lotion exhibited a notable emission of C_8_H_10_O_2_, likely phenoxyethanol, a fragrant compound commonly used as a preservative in cosmetics.^39,40^ The VOC emission dynamics from the “spray-type” products are different from the roll-on deodorant and hand lotion. The use of “spray-type” products resulted in a pulse release of VOCs, followed by a first-order decay pattern. In contrast, using the roll-on deodorant and hand lotion led to relatively stable VOC emissions throughout the 1 h emission period.

Significant emissions of terpenes and their derivatives from the PCPs were observed (Figure 1B). Monoterpenes were emitted from all five products, with peak concentrations ranging from approximately 0.06 to 37 ppb following product use. TD-GC-MS analysis identified several emitted monoterpenes, including β-myrcene, α-pinene, β-pinene, limonene, and γ-terpinene. Additionally, emissions of monoterpenoids were also observed, and some of them were identified by TD-GC-MS analysis, including C_10_H_16_O, C_10_H_18_O (eucalyptol and linalool), C_10_H_20_O (citronellol and dihydro-α-terpineol), and C_12_H_20_O_2_ (α-terpinyl acetate and linalyl acetate). Beyond the commonly identified fragrances in previous PCP studies, we discovered remarkable emissions of sesquiterpenes (C_15_H_24_) and several terpene-related chemicals with a carbon number greater than 13, such as C_13_H_20_O, C_14_H_22_O, C_14_H_24_O_2_, C_16_H_26_O, and C_17_H_26_O. Sesquiterpenes are very sensitive components of particle production rates as they are efficient precursors of ultralow volatility organic compounds upon ozone-initiated oxidation.^41^ The concentration of sesquiterpenes, C_16_H_26_O, and C_17_H_26_O could reach 0.3–0.7 ppb and 1.3–4.3 ppb after spraying the perfume and body spray, respectively. Even though their identities cannot be obtained through the TD-GC-MS analysis, C_13_H_20_O, C_14_H_22_O, C_16_H_26_O, and C_17_H_26_O are potentially associated with ionone, α-isomethyl ionone, tetramethyl acetyloctahydronaphthalenes (OTNE), and acetyl cedrene, respectively, which are fragrant chemicals commonly used in perfume and cosmetic products.^42,43^ A notable feature of the terpenes and their derivatives found in this study is that the majority of them contain unsaturated C=C double bonds, which may readily react with indoor gas-phase oxidants, such as ozone. Even though we were not able to determine the concentrations of some of the individual terpenes and derivatives due to the existence of isomers, linalool and β-myrcene are the two compounds exhibiting the highest rate coefficients among the aforementioned compounds for reactions of O_3_ (Table S2; 4.1 × 10^–16^ and 4.7 × 10^–16^ cm^3^ molec.^–1^ s^–1^), which are important in terms of gas-phase ozone loss.

**Figure 2:**
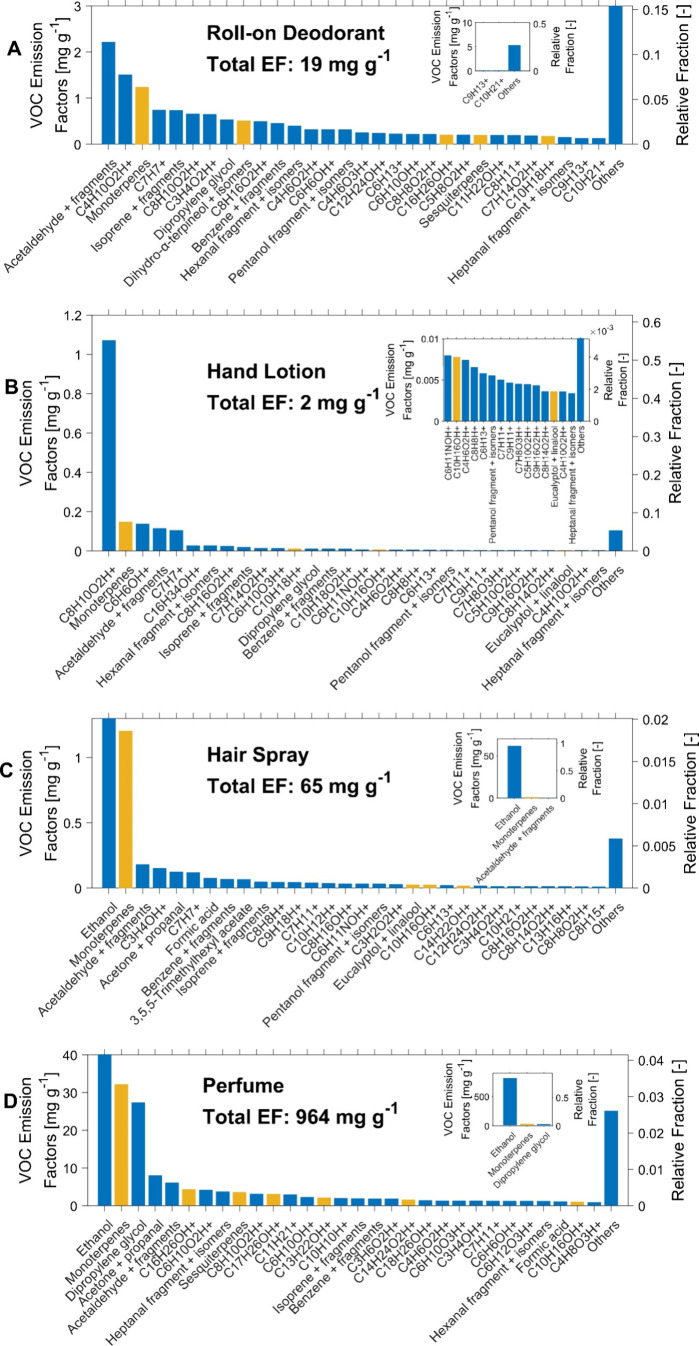

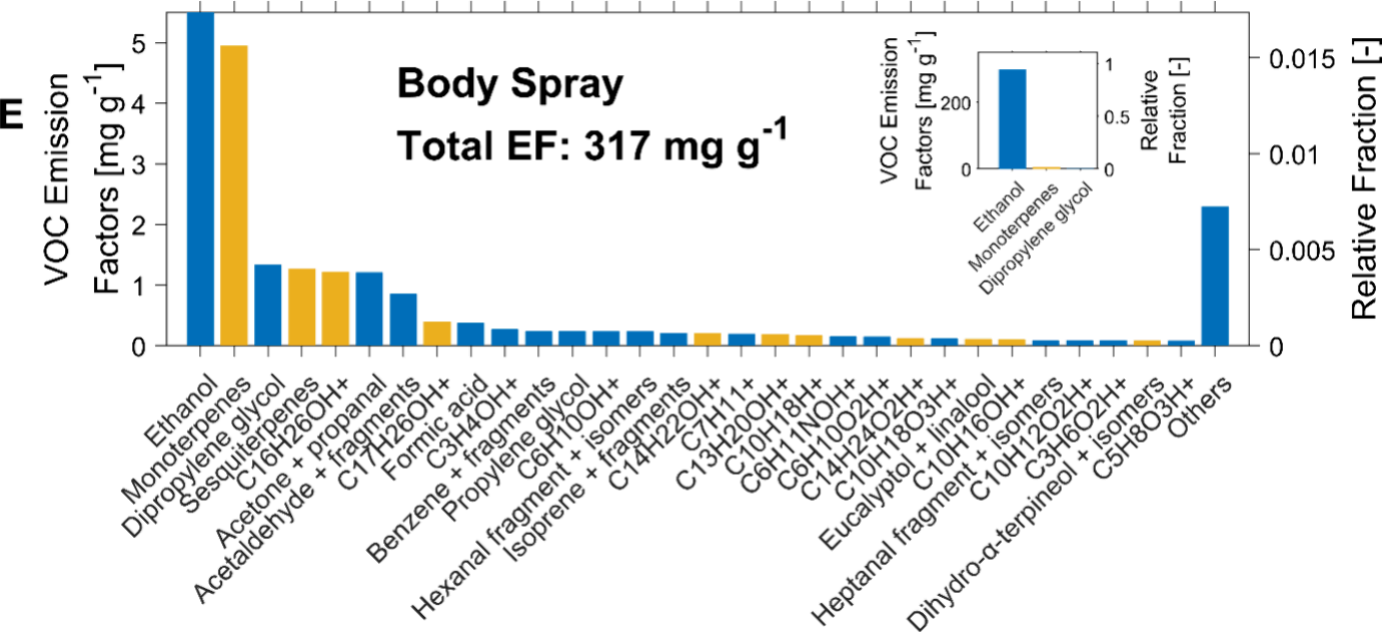
VOC emission factors (EFs) of the tested PCPs, estimated from the measurements of the Vocus PTR in the primary emission experiments. The yellow bars indicate that the compounds may contain unsaturated C=C double bonds and their oxidation products have been detected. The assignment of the species was based on the offline VOC analysis with the TD-GC-MS and previous literature on indoor air measurements with a proton-transfer-reaction mass spectrometer (PTR-MS).

Gas-phase VOC emission factors (EFs) for each tested PCP were estimated based on measurements obtained with the Vocus PTR during the primary emission experiments (Figure 2). The EF of a VOC represents the ratio of the emitted mass of the VOC to the mass consumption of the product of the use (details in the SI). It is important to note that the Kimwipes and glass plates used for the roll-on deodorant and hand lotion were only placed in the chamber for 1 h during the experiments and the glass plates were not heated to body temperature. As a result, their EFs solely represent the first-hour emissions and serve as the lower bound of the actual EF. The perfume exhibited the highest total EF of 964 mg g^–1^, indicating that the liquid consists predominantly of VOCs in ethanol and evaporates rapidly after use. The body spray showed the second-highest total EF (318 mg g^–1^), followed by hair spray (65 mg g^–1^), roll-on deodorant (19 mg g^–1^), and hand lotion (2 mg g^–1^). Ethanol was the dominant VOC emitted from the hair spray, perfume, and body spray, accounting for 95%, 84%, and 95% of the total EF, respectively. The total EF of the hand lotion and roll-on deodorant was dominated by C_8_H_10_O_2_ (tentatively phenoxyethanol) and C_2_H_4_O (acetaldehyde), respectively. The monoterpene EFs of roll-on deodorant, hand lotion, hair spray, perfume, and body spray were 1241, 148, 1204, 32219, and 4953 μg g^–1^, respectively. They exhibit the second-highest EF in hand lotion, perfume, hair spray, and body spray, and the third-highest EF in the roll-on deodorant. Interestingly, none of the tested PCPs emitted cyclic methyl siloxanes (Figure S1), which are a class of VOCs found in multiple indoor air studies originating from PCPs.^46,47^

It is important to acknowledge that the reported EFs only account for VOCs with a proton affinity greater than water, detectable by the Vocus PTR. However, the PCPs may also emit alkanes, which possess a proton affinity less than water and are therefore undetectable. For example, the ingredient chart of the hair spray includes propane and butane (Table S3), commonly used in many pressurized spray cans but not detectable by the Vocus PTR with hydronium (H_3_O^+^) primary reagent ions. Additionally, the Vocus PTR may not be sensitive to certain organic compounds emitted from the PCPs if they undergo fragmentation in the focusing ion molecule reactor (FIMR) or adhere to inlet tubing walls. During the primary emission experiments with the I-CIMS, we identified the emission of several organic compounds with a carbon number greater than 15 from the body spray, for example, C_15_H_28_O_5_, C_20_H_40_O, C_20_H_42_O, C_21_H_44_O, C_22_H_46_O, which were not detected by the Vocus PTR (Figure S2). They have not been reported in previous indoor air studies. C_20_H_42_O, C_20_H_40_O, C_21_H_44_O, and C_22_H_46_O may be associated with octyldodecanol, phytol, 1-heneicosanol, 1-docosanol, respectively, which are used as fragrant additives in perfume or serve as emulsifier, emollient, thickener in cosmetics.^48−50^ It should be noted that the VOC EFs reported in this study may represent the lower bound of the actual EF due to potential limitations in the detection capabilities of the instruments.

### Particle Formation and Dynamics

When using the tested PCPs with elevated indoor ozone levels, rapid new particle formation (NPF) events were observed, except when applying the roll-on deodorant. Figure 1C presents a time-series plot of aerosol size distribution in an oxidation experiment, exhibiting four clear “banana” curves. Aerosol nucleation was observed immediately after the use of the hand lotion, hair spray, perfume, and body spray. Then the nucleated aerosols grew fast and led to the significant formation of ultrafine particles (UFPs; < 100 nm). The particle growth rate^51^ from 3 to 7 nm ranged between 30 and 40 nm h^–1^, and from 7 to 20 nm between 15 and 23 nm h^–1^, which are considerably higher than those reported in atmospheric aerosol studies in urban or remote environments (Table S4). The peak number concentrations of UFPs during the four NPF events ranged from ∼34000 to ∼200000 cm^–3^, surpassing the UFP concentrations in many urban atmospheric environments.^44,52^ The NPF events dramatically elevated indoor PM_1_ concentrations (mass concentration of all the aerosols with an aerodynamic diameter smaller than 1 μm). In the last experiment with body spray, the PM_1_ concentration exceeded 20 μg m^–3^, which was three times higher than the previously reported annual average PM_1_ concentration in urban Zürich.^45^

The variation of the SOA formation potential among different PCPs was attributable to the differences in VOC precursors, their emission rates, and emission factors. The formation potential is also influenced by oxidant concentration, NO_*x*_ concentration, and environmental conditions, such as AER, since they can affect the abundance of precursor VOCs, the formation rate of condensable vapors, room air retention time, and aerosol condensation sink. In a supplementary oxidation experiment with a lower ozone concentration (25–30 ppb) and a higher AER (1.83 h^–1^), the PM_1_ mass concentrations in the experiments for perfume, hair spray, and body spray were lower by more than 50% (Figure S2) than those in the experiments shown in Figure 1, with even greater consumption of the PCPs (Figure S2), as the formation rate of condensable vapors and condensable aerosol surface area were lower, leading to lower particle mass concentrations.

### Gas-Phase Oxidation Products

The oxidation experiments revealed a wide range of gas-phase oxidized organic compounds with a varying number of carbon elements (1 to 17) and oxygen elements (up to 8) (Figures S4 and S5). The concentration profiles of the oxidation products follow the use of the PCPs (Figure 1E-F and Figure S2C–I). Among the oxidation products, C_10_H_16_O_3_, CH_4_O_3_ (tentatively hydroxymethyl hydroperoxide), and C_9_H_14_O_5_ exhibited the highest signal intensity. We speculated that the oxidation products were primarily formed through the ozonolysis of terpenes and their derivatives that are used as fragrant additives in the PCPs. Additionally, we also observed several N-containing oxidation products (e.g., C_10_H_17_NO_5–7_) in a further supplementary oxidation experiment with the I-CIMS (Figure S2H), which may have formed through nitrate-radical-induced autoxidation, as we observed the formation of N_2_O_5_ with the I-CIMS in the chamber before the experiment.^53,54^

Monoterpenes are the dominant reactive VOCs emitted from the PCPs with respect to ozone. Given the highest EFs of monoterpenes among all the reactive VOCs that are emitted from the PCPs and their high reaction rate coefficients with ozone (Table S2), we speculated that monoterpenes were the most consumed VOCs in the gas-phase reactions. Figure 1E and Figure S2C–F show the abundance of monoterpene oxidation products measured with the Vocus PTR and I-CIMS, respectively. Many of these compounds have been reported in field measurements and chamber experiments in atmospheric chemistry studies, such as C_10_H_16_O_*x*_, C_9_H_12_O_*x*_, C_9_H_14_O_*x*_, and C_8_H_12_O_*x*_, as indicated in the mass defect plot of organic vapors measured by the Vocus PTR and I-CIMS (Figure S3). It is known that ozonolysis of monoterpene produces highly oxygenated organic molecules (HOMs), which play an important role in aerosol nucleation and growth in outdoor environments.^55−58^ Despite not being effectively detected by the Vocus PTR and I-CIMS, we speculate based on experiments reported in the literature that HOMs with more than 8 oxygen atoms will have formed, and together with the detected low-volatility monoterpene oxidation products, significantly contribute to particle formation.

Aside from monoterpene oxidation products, we found that sesquiterpene and other terpenoids from the PCPs also underwent oxidation reactions, leading to multiple highly oxygenated oxidation products not commonly reported in previous studies. C_10_H_18_O_4–6_, C_10_H_20_O_3–6_, C_12_H_20_O_3–7_, C_14_H_22_O_4–5_, and C_15_H_24_O_3–5_ (Figure 1F and Figure S2G–H) might be associated with the oxidation of linalool, citronellol, α-terpinyl acetate, linalyl acetate, and sesquiterpenes, which were identified in the primary emission experiments.^59−63^ Moreover, newly identified compounds, such as C_13_H_20_O_3–5_, C_16_H_26_O_3–4_, and C_17_H_26_O_3–4_, found in the perfume and body spray oxidation experiments, are hypothesized to be produced from the oxidation of C_13_H_20_O (tentatively ionone), C_16_H_26_O (tentatively OTNE), and C_17_H_26_O (tentatively α-isomethyl ionone), respectively. These oxidation products are classified as low-volatility organic compounds and semivolatile organic compounds (Figure S4), and they also could contribute significantly to the formation and growth of particles in PCP oxidation experiments.^64^

## Supporting Information Available

Detailed description of study site, experimental methods, and measurements (along with Figure S9); details on calculation of VOC emission factors (along with Figures S7–8); discussion on VOC oxidation by hydroxyl and nitrate radicals; comparison with atmospheric VOC emission inventories; further discussion on implications for indoor air quality and study limitations; supplement results from primary and oxidation experiments (Tables S1–5; Figures S1–5); characterization of background level of particles and VOCs (Figures S6, S10, and S11) (PDF)

## References

[ref1] Daughton C. G.; Ternes T. A. Pharmaceuticals and Personal Care Products in the Environment: Agents of Subtle Change?. Environ. Health Perspect 1999, 107 (Suppl 6), 907–938. 10.1289/ehp.99107s6907. 10592150 PMC1566206

[ref2] Ebele A. J.; Abou-Elwafa Abdallah M.; Harrad S. Pharmaceuticals and Personal Care Products (PPCPs) in the Freshwater Aquatic Environment. Emerg Contam 2017, 3 (1), 1–16. 10.1016/j.emcon.2016.12.004.

[ref3] Westerhoff P.; Yoon Y.; Snyder S.; Wert E. Fate of Endocrine-Disruptor, Pharmaceutical, and Personal Care Product Chemicals during Simulated Drinking Water Treatment Processes. Environ. Sci. Technol. 2005, 39 (17), 6649–6663. 10.1021/es0484799. 16190224

[ref4] Steinemann A. Volatile Emissions from Common Consumer Products. Air Qual Atmos Health 2015, 8 (3), 273–281. 10.1007/s11869-015-0327-6.

[ref5] Nematollahi N.; Kolev S. D.; Steinemann A. Volatile Chemical Emissions from 134 Common Consumer Products. Air Qual Atmos Health 2019, 12, 1259–1265. 10.1007/s11869-019-00754-0.

[ref6] Nematollahi N.; Doronila A.; Mornane P. J.; Duan A.; Kolev S. D.; Steinemann A. Volatile Chemical Emissions from Fragranced Baby Products. Air Qual Atmos Health 2018, 11, 785–790. 10.1007/s11869-018-0593-1. 30147808 PMC6097056

[ref7] Yeoman A. M.; Shaw M.; Lewis A. C. Estimating Person-to-person Variability in VOC Emissions from Personal Care Products Used during Showering. Indoor Air 2021, 31 (4), 1281–1291. 10.1111/ina.12811. 33615569

[ref8] Goodman N.; Nematollahi N. Fragranced Consumer Products as Sources. In Handbook of Indoor Air Quality; Springer, 2022; pp 129–161.

[ref9] Lee A.; Goldstein A. H.; Kroll J. H.; Ng N. L.; Varutbangkul V.; Flagan R. C.; Seinfeld J. H. Gas-phase Products and Secondary Aerosol Yields from the Photooxidation of 16 Different Terpenes. Journal of Geophysical Research: Atmospheres 2006, 111 (D17), D17305 10.1029/2006JD007050.

[ref10] Larsen B. R.; Di Bella D.; Glasius M.; Winterhalter R.; Jensen N. R.; Hjorth J. Gas-Phase OH Oxidation of Monoterpenes: Gaseous and Particulate Products. J. Atmos Chem. 2001, 38, 231–276. 10.1023/A:1006487530903.

[ref11] Yu J.; Cocker D. R.; Griffin R. J.; Flagan R. C.; Seinfeld J. H. Gas-Phase Ozone Oxidation of Monoterpenes: Gaseous and Particulate Products. J. Atmos Chem. 1999, 34, 207–258. 10.1023/A:1006254930583.

[ref12] Zhang H.; Yee L. D.; Lee B. H.; Curtis M. P.; Worton D. R.; Isaacman-VanWertz G.; Offenberg J. H.; Lewandowski M.; Kleindienst T. E.; Beaver M. R.; et al. Monoterpenes Are the Largest Source of Summertime Organic Aerosol in the Southeastern United States. Proc. Natl. Acad. Sci. U. S. A. 2018, 115 (9), 2038–2043. 10.1073/pnas.1717513115. 29440409 PMC5834703

[ref13] Huang H.-L.; Tsai T.-J.; Hsu N.-Y.; Lee C.-C.; Wu P.-C.; Su H.-J. Effects of Essential Oils on the Formation of Formaldehyde and Secondary Organic Aerosols in an Aromatherapy Environment. Build Environ 2012, 57, 120–125. 10.1016/j.buildenv.2012.04.020.

[ref14] Singer B. C.; Coleman B. K.; Destaillats H.; Hodgson A. T.; Lunden M. M.; Weschler C. J.; Nazaroff W. W. Indoor Secondary Pollutants from Cleaning Product and Air Freshener Use in the Presence of Ozone. Atmos. Environ. 2006, 40 (35), 6696–6710. 10.1016/j.atmosenv.2006.06.005. 16903280

[ref15] Rosales C. M. F.; Jiang J.; Lahib A.; Bottorff B. P.; Reidy E. K.; Kumar V.; Tasoglou A.; Huber H.; Dusanter S.; Tomas A.; et al. Chemistry and Human Exposure Implications of Secondary Organic Aerosol Production from Indoor Terpene Ozonolysis. Sci. Adv. 2022, 8 (8), eabj9156 10.1126/sciadv.abj9156. 35213219 PMC8880786

[ref16] Khan F.; Kwapiszewska K.; Zhang Y.; Chen Y.; Lambe A. T.; Kołodziejczyk A.; Jalal N.; Rudzinski K.; Martínez-Romero A.; Fry R. C.; et al. Toxicological Responses of α-Pinene-Derived Secondary Organic Aerosol and Its Molecular Tracers in Human Lung Cell Lines. Chem. Res. Toxicol. 2021, 34 (3), 817–832. 10.1021/acs.chemrestox.0c00409. 33653028 PMC7967287

[ref17] Nørgaard A. W.; Kudal J. D.; Kofoed-Sørensen V.; Koponen I. K.; Wolkoff P. Ozone-Initiated VOC and Particle Emissions from a Cleaning Agent and an Air Freshener: Risk Assessment of Acute Airway Effects. Environ. Int. 2014, 68, 209–218. 10.1016/j.envint.2014.03.029. 24769411

[ref18] Shiraiwa M.; Ueda K.; Pozzer A.; Lammel G.; Kampf C. J.; Fushimi A.; Enami S.; Arangio A. M.; Fröhlich-Nowoisky J.; Fujitani Y.; Furuyama A.; Lakey P. S. J.; Lelieveld J.; Lucas K.; Morino Y.; Pöschl U.; Takahama S.; Takami A.; Tong H.; Weber B.; Yoshino A.; Sato K. Aerosol Health Effects from Molecular to Global Scales. Environ. Sci. Technol. 2017, 51, 13545–13567. 10.1021/acs.est.7b04417. 29111690

[ref19] Wu T.; Tasoglou A.; Wagner D. N.; Jiang J.; Huber H. J.; Stevens P. S.; Jung N.; Boor B. E. Modern Buildings Act as a Dynamic Source and Sink for Urban Air Pollutants. Cell Reports Sustainability 2024, 1 (5), 100103 10.1016/j.crsus.2024.100103.

[ref20] Gkatzelis G. I.; Coggon M. M.; McDonald B. C.; Peischl J.; Gilman J. B.; Aikin K. C.; Robinson M. A.; Canonaco F.; Prevot A. S. H.; Trainer M.; et al. Observations Confirm That Volatile Chemical Products Are a Major Source of Petrochemical Emissions in US Cities. Environ. Sci. Technol. 2021, 55 (8), 4332–4343. 10.1021/acs.est.0c05471. 33720711

[ref21] Sheu R.; Fortenberry C. F.; Walker M. J.; Eftekhari A.; Stönner C.; Bakker A.; Peccia J.; Williams J.; Morrison G. C.; Williams B. J.; et al. Evaluating Indoor Air Chemical Diversity, Indoor-to-Outdoor Emissions, and Surface Reservoirs Using High-Resolution Mass Spectrometry. Environ. Sci. Technol. 2021, 55 (15), 10255–10267. 10.1021/acs.est.1c01337. 34270218 PMC8461992

[ref22] Coggon M. M.; McDonald B. C.; Vlasenko A.; Veres P. R.; Bernard F.; Koss A. R.; Yuan B.; Gilman J. B.; Peischl J.; Aikin K. C.; et al. Diurnal Variability and Emission Pattern of Decamethylcyclopentasiloxane (D5) from the Application of Personal Care Products in Two North American Cities. Environ. Sci. Technol. 2018, 52 (10), 5610–5618. 10.1021/acs.est.8b00506. 29659257

[ref23] Gkatzelis G. I.; Coggon M. M.; McDonald B. C.; Peischl J.; Aikin K. C.; Gilman J. B.; Trainer M.; Warneke C. Identifying Volatile Chemical Product Tracer Compounds in US Cities. Environ. Sci. Technol. 2021, 55 (1), 188–199. 10.1021/acs.est.0c05467. 33325693

[ref24] Coggon M. M.; Gkatzelis G. I.; McDonald B. C.; Gilman J. B.; Schwantes R. H.; Abuhassan N.; Aikin K. C.; Arend M. F.; Berkoff T. A.; Brown S. S. Volatile Chemical Product Emissions Enhance Ozone and Modulate Urban Chemistry. Proc. Natl. Acad. Sci. U. S. A. 2021, 118 (32), e2026653118 10.1073/pnas.2026653118. 34341119 PMC8364211

[ref25] McDonald B. C.; De Gouw J. A.; Gilman J. B.; Jathar S. H.; Akherati A.; Cappa C. D.; Jimenez J. L.; Lee-Taylor J.; Hayes P. L.; McKeen S. A.; et al. Volatile Chemical Products Emerging as Largest Petrochemical Source of Urban Organic Emissions. Science (1979) 2018, 359 (6377), 760–764. 10.1126/science.aaq0524. 29449485

[ref26] Alton M. W.; Browne E. C. Atmospheric Degradation of Cyclic Volatile Methyl Siloxanes: Radical Chemistry and Oxidation Products. ACS Environ. Au 2022, 2 (3), 263–274. 10.1021/acsenvironau.1c00043. 37102141 PMC10114625

[ref27] Sasidharan S.; He Y.; Akherati A.; Li Q.; Li W.; Cocker D.; McDonald B. C.; Coggon M. M.; Seltzer K. M.; Pye H. O. T.; et al. Secondary Organic Aerosol Formation from Volatile Chemical Product Emissions: Model Parameters and Contributions to Anthropogenic Aerosol. Environ. Sci. Technol. 2023, 57 (32), 11891–11902. 10.1021/acs.est.3c00683. 37527511 PMC11610419

[ref28] Pennington E. A.; Seltzer K. M.; Murphy B. N.; Qin M.; Seinfeld J. H.; Pye H. O. T. Modeling Secondary Organic Aerosol Formation from Volatile Chemical Products. Atmos Chem. Phys. 2021, 21 (24), 18247–18261. 10.5194/acp-21-18247-2021. 35087576 PMC8788583

[ref29] Milani A.; Al-Naiema I. M.; Stone E. A. Detection of a Secondary Organic Aerosol Tracer Derived from Personal Care Products. Atmos. Environ. 2021, 246, 118078 10.1016/j.atmosenv.2020.118078.

[ref30] Charan S. M.; Huang Y.; Buenconsejo R. S.; Li Q.; Cocker III D. R.; Seinfeld J. H. Secondary Organic Aerosol Formation from the Oxidation of Decamethylcyclopentasiloxane at Atmospherically Relevant OH Concentrations. Atmos Chem. Phys. 2022, 22 (2), 917–928. 10.5194/acp-22-917-2022.

[ref31] Janechek N. J.; Hansen K. M.; Stanier C. O. Comprehensive Atmospheric Modeling of Reactive Cyclic Siloxanes and Their Oxidation Products. Atmos Chem. Phys. 2017, 17 (13), 8357–8370. 10.5194/acp-17-8357-2017. 30740128 PMC6368090

[ref32] Janechek N. J.; Marek R. F.; Bryngelson N.; Singh A.; Bullard R. L.; Brune W. H.; Stanier C. O. Physical Properties of Secondary Photochemical Aerosol from OH Oxidation of a Cyclic Siloxane. Atmos Chem. Phys. 2019, 19 (3), 1649–1664. 10.5194/acp-19-1649-2019. 31889955 PMC6936766

[ref33] ACGIH. Ethanol, TLV. https://www.acgih.org/ethanol/. (accessed 2024-08-05).

[ref34] Gottschalck T. E. International Cosmetic Ingredient Dictionary and Handbook; CD-ROM. Scientific Regulatory 2006 Reference CD; Cosmetic, Toiletry, and Fragrance Assoc., 2006.

[ref35] Arctander S. Perfume & Flavor Chemicals (Aroma Chemicals), Vol. II; Lulu, 2019.

[ref36] vom Ende M.; Sturm W.; Peters K. Ullmann’s Encyclopedia of Industrial Chemistry, Perfumes, 7th ed.; Wiley, 2017. 10.1002/14356007.a19_171.pub2.

[ref37] Teixeira M. A.; Rodríguez O.; Mata V. G.; Rodrigues A. E. The Diffusion of Perfume Mixtures and the Odor Performance. Chem. Eng. Sci. 2009, 64 (11), 2570–2589. 10.1016/j.ces.2009.01.064.

[ref38] Api A. M.; Belsito D.; Botelho D.; Browne D.; Bruze M.; Burton G. A. Jr; Buschmann J.; Dagli M. L.; Date M.; Dekant W.; et al. RIFM Fragrance Ingredient Safety Assessment, Octanal Dimethyl Acetal, CAS Registry Number 10022-28-3. Food Chem. Toxicol. 2018, 115, S225–S230. 10.1016/j.fct.2018.01.039. 29396138

[ref39] Akgündüz M. C.; Çavuşoğlu K.; Yalçın E. The Potential Risk Assessment of Phenoxyethanol with a Versatile Model System. Sci. Rep 2020, 10 (1), 1209 10.1038/s41598-020-58170-9. 31988350 PMC6985251

[ref40] Scognamiglio J.; Jones L.; Letizia C. S.; Api A. M. Fragrance Material Review on 2-Phenoxyethanol. Food and chemical toxicology 2012, 50, S244–S255. 10.1016/j.fct.2011.10.030. 22036980

[ref41] Dada L.; Stolzenburg D.; Simon M.; Fischer L.; Heinritzi M.; Wang M.; Xiao M.; Vogel A. L.; Ahonen L.; Amorim A.; et al. Role of Sesquiterpenes in Biogenic New Particle Formation. Sci. Adv. 2023, 9 (36), eadi5297 10.1126/sciadv.adi5297. 37682996 PMC10491295

[ref42] Guć M.; Cegłowski M.; Pawlaczyk M.; Kurczewska J.; Reszke E.; Schroeder G. Application of FAPA Mass Spectrometry for Analysis of Fragrance Ingredients Used in Cosmetics. Measurement 2021, 168, 108326 10.1016/j.measurement.2020.108326.

[ref43] Scognamiglio J.; Letizia C. S.; Politano V. T.; Api A. M. Fragrance Material Review on Acetyl Cedrene. Food and chemical toxicology 2013, 62, S152–S166. 10.1016/j.fct.2013.07.053. 23907023

[ref44] Zhou L.; Kim E.; Hopke P. K.; Stanier C. O.; Pandis S. Advanced Factor Analysis on Pittsburgh Particle Size-Distribution Data. Aerosol Sci. Technol. 2004, 38 (Suppl 1), 118–132. 10.1080/02786820390229589.

[ref45] Chen G.; Canonaco F.; Tobler A.; Aas W.; Alastuey A.; Allan J.; Atabakhsh S.; Aurela M.; Baltensperger U.; Bougiatioti A.; et al. European Aerosol Phenomenology– 8: Harmonised Source Apportionment of Organic Aerosol Using 22 Year-Long ACSM/AMS Datasets. Environ. Int. 2022, 166, 107325 10.1016/j.envint.2022.107325. 35716508

[ref46] Tang X.; Misztal P. K.; Nazaroff W. W.; Goldstein A. H. Volatile Organic Compound Emissions from Humans Indoors. Environ. Sci. Technol. 2016, 50 (23), 12686–12694. 10.1021/acs.est.6b04415. 27934268

[ref47] Tang X.; Misztal P. K.; Nazaroff W. W.; Goldstein A. H. Siloxanes Are the Most Abundant Volatile Organic Compound Emitted from Engineering Students in a Classroom. Environ. Sci. Technol. Lett. 2015, 2 (11), 303–307. 10.1021/acs.estlett.5b00256.

[ref48] Belsito D.; Bickers D.; Bruze M.; Calow P.; Greim H.; Hanifin J. M.; Rogers A. E.; Saurat J. H.; Sipes I. G.; Tagami H. A Safety Assessment of Non-Cyclic Alcohols with Unsaturated Branched Chain When Used as Fragrance Ingredients: The RIFM Expert Panel. Food Chem. Toxicol. 2010, 48, S1–S42. 10.1016/j.fct.2009.11.007. 20141871

[ref49] McGinty D.; Letizia C. S.; Api A. M. Fragrance Material Review on Isophytol. Food and chemical toxicology 2010, 48, S76–S81. 10.1016/j.fct.2009.11.015. 20141882

[ref50] Office of Pollution Prevention and Toxics. Supporting Information for Low-Priority Substance 1-Docosanol (CASRN 661-19-8) Final Designation; Washington, DC, 2020.

[ref51] Dada L.; Lehtipalo K.; Kontkanen J.; Nieminen T.; Baalbaki R.; Ahonen L.; Duplissy J.; Yan C.; Chu B.; Petäjä T.; Lehtinen K.; Kerminen V.-M.; Kulmala M.; Kangasluoma J. Formation and Growth of Sub-3-Nm Aerosol Particles in Experimental Chambers. Nat. Protoc 2020, 15 (3), 1013–1040. 10.1038/s41596-019-0274-z. 32051616

[ref52] Kumar P.; Morawska L.; Birmili W.; Paasonen P.; Hu M.; Kulmala M.; Harrison R. M.; Norford L.; Britter R. Ultrafine Particles in Cities. Environ. Int. 2014, 66, 1–10. 10.1016/j.envint.2014.01.013. 24503484

[ref53] Lee B. H.; Mohr C.; Lopez-Hilfiker F. D.; Lutz A.; Hallquist M.; Lee L.; Romer P.; Cohen R. C.; Iyer S.; Kurtén T.; et al. Highly Functionalized Organic Nitrates in the Southeast United States: Contribution to Secondary Organic Aerosol and Reactive Nitrogen Budgets. Proc. Natl. Acad. Sci. U. S. A. 2016, 113 (6), 1516–1521. 10.1073/pnas.1508108113. 26811465 PMC4760802

[ref54] Dam M.; Draper D. C.; Marsavin A.; Fry J. L.; Smith J. N. Observations of Gas-Phase Products from the Nitrate-Radical-Initiated Oxidation of Four Monoterpenes. Atmos Chem. Phys. 2022, 22 (13), 9017–9031. 10.5194/acp-22-9017-2022.

[ref55] Bianchi F.; Kurten T.; Riva M.; Mohr C.; Rissanen M. P.; Roldin P.; Berndt T.; Crounse J. D.; Wennberg P. O.; Mentel T. F.; Wildt J.; Junninen H.; Jokinen T.; Kulmala M.; Worsnop D. R.; Thornton J. A.; Donahue N.; Kjaergaard H. G.; Ehn M. Highly Oxygenated Organic Molecules (HOM) from Gas-Phase Autoxidation Involving Peroxy Radicals : A Key Contributor to Atmospheric Aerosol. Chem. Rev. 2019, 119, 3472 10.1021/acs.chemrev.8b00395. 30799608 PMC6439441

[ref56] Ehn M.; Thornton J. A.; Kleist E.; Sipilä M.; Junninen H.; Pullinen I.; Springer M.; Rubach F.; Tillmann R.; Lee B.; Lopez-Hilfiker F.; Andres S.; Acir I. H.; Rissanen M.; Jokinen T.; Schobesberger S.; Kangasluoma J.; Kontkanen J.; Nieminen T.; Kurtén T.; Nielsen L. B.; Jørgensen S.; Kjaergaard H. G.; Canagaratna M.; Maso M. D.; Berndt T.; Petäjä T.; Wahner A.; Kerminen V. M.; Kulmala M.; Worsnop D. R.; Wildt J.; Mentel T. F. A Large Source of Low-Volatility Secondary Organic Aerosol. Nature 2014, 506 (7489), 476–479. 10.1038/nature13032. 24572423

[ref57] Pospisilova V.; Lopez-Hilfiker F. D.; Bell D. M.; El Haddad I.; Mohr C.; Huang W.; Heikkinen L.; Xiao M.; Dommen J.; Prevot A. S. H.; et al. On the Fate of Oxygenated Organic Molecules in Atmospheric Aerosol Particles. Sci. Adv. 2020, 6 (11), eaax8922 10.1126/sciadv.aax8922. 32201715 PMC7069715

[ref58] Roldin P.; Ehn M.; Kurtén T.; Olenius T.; Rissanen M. P.; Sarnela N.; Elm J.; Rantala P.; Hao L.; Hyttinen N.; et al. The Role of Highly Oxygenated Organic Molecules in the Boreal Aerosol-Cloud-Climate System. Nat. Commun. 2019, 10 (1), 4370 10.1038/s41467-019-12338-8. 31554809 PMC6761173

[ref59] Elsharif S. A.; Banerjee A.; Buettner A. Structure-Odor Relationships of Linalool, Linalyl Acetate and Their Corresponding Oxygenated Derivatives. Front Chem. 2015, 3, 57 10.3389/fchem.2015.00057. 26501053 PMC4594031

[ref60] Kern S.; Dkhil H.; Hendarsa P.; Ellis G.; Natsch A. Detection of Potentially Skin Sensitizing Hydroperoxides of Linalool in Fragranced Products. Anal Bioanal Chem. 2014, 406, 6165–6178. 10.1007/s00216-014-8066-3. 25138721

[ref61] Shu Y.; Kwok E. S. C.; Tuazon E. C.; Atkinson R.; Arey J. Products of the Gas-Phase Reactions of Linalool with OH Radicals, NO3 Radicals, and O3. Environ. Sci. Technol. 1997, 31 (3), 896–904. 10.1021/es960651o.

[ref62] Barreira L. M. F.; Ylisirniö A.; Pullinen I.; Buchholz A.; Li Z.; Lipp H.; Junninen H.; Hõrrak U.; Noe S. M.; Krasnova A.; et al. The Importance of Sesquiterpene Oxidation Products for Secondary Organic Aerosol Formation in a Springtime Hemiboreal Forest. Atmos Chem. Phys. 2021, 21 (15), 11781–11800. 10.5194/acp-21-11781-2021.

[ref63] Yee L. D.; Isaacman-VanWertz G.; Wernis R. A.; Meng M.; Rivera V.; Kreisberg N. M.; Hering S. V.; Bering M. S.; Glasius M.; Upshur M. A.; et al. Observations of Sesquiterpenes and Their Oxidation Products in Central Amazonia during the Wet and Dry Seasons. Atmos Chem. Phys. 2018, 18 (14), 10433–10457. 10.5194/acp-18-10433-2018. 33354203 PMC7751628

[ref64] Donahue N. M.; Kroll J. H.; Pandis S. N.; Robinson A. L. A Two-Dimensional Volatility Basis Set–Part 2: Diagnostics of Organic-Aerosol Evolution. Atmos Chem. Phys. 2012, 12 (2), 615–634. 10.5194/acp-12-615-2012.

