# Supplementary material for: Indoor Emission, Oxidation, and New Particle Formation of Personal Care Product Related Volatile Organic Compounds
Source: Environ Sci Technol Lett. 2024 Aug 30;11(10):1053–61. doi: 10.1021/acs.estlett.4c00353 (PMC11465640; doi:10.1021/acs.estlett.4c00353)
Supplement: Supplementary file 1 — ez4c00353_si_001.pdf [file ez4c00353_si_001.pdf]

Supporting Information for: Indoor Emission, Oxidation, and New Particle Formation of Personal Care Product Related Volatile Organic Compounds

Tianren Wu[1,2], Tatjana Müller[3], Nijing Wang[3], Joseph Byron[3], Sarka Langer[4,5], Jonathan Williams[3], Dusan Licina[1]*

[1]Human-Oriented Built Environment Lab, School of Architecture, Civil and Environmental Engineering, École Polytechnique Fédérale de Lausanne, CH-1015 Lausanne, Switzerland

[2]Civil and Architectural Engineering and Construction Management, University of Cincinnati, Cincinnati, OH, USA 45221

[3]Atmospheric Chemistry Department, Max Planck Institute for Chemistry, Hahn-Meitner-Weg 1, 55128 Mainz, Germany

[4]IVL Swedish Environmental Research Institute, Environmental Chemistry, SE-400 14 Göteborg, Sweden

[5]Chalmers University of Technology, Department of Architecture and Civil Engineering, Division of Building Services Engineering, SE-412 96 Göteborg, Sweden

*Corresponding author: e-mail: dusan.licina@epfl.ch

Table of Contents

Number of Pages: 23

Number of Figures: 11

Number of Tables: 5

Table of Contents:

Detailed Description of Study Site, Experimental Methods, and Measurements: pg. S2-S4

Uncertainties Regarding Quantifying Ethanol with the Vocus PTR: pg. S4

Details on Calculation of VOC Emission Factors (EFs): pg. S4-S5

VOC Oxidation by Hydroxyl and Nitrate Radicals: pg. S5

Comparison with Atmospheric VOC Emission Inventories: pg. S5

Implications for Indoor Air Quality and Study Limitations: pg. S6

Tables (S1-S5): pg. S8-S13

Figures (S1-S11): pg. S13-S19

References: pg. S20-24

Detailed Description of Study Site, Experimental Methods, and Measurements

The indoor environmental chamber represents a modern office environment with an area of 24.7 m^2 and a volume of 62 m^3. There were three office chairs and desks in the chamber. The chamber has painted walls, hard floors, and metallic ceiling panels. In all experiments, the chamber was positively pressurized, with the supply air flow rate ~10% higher than the return air flow rate, to prevent infiltration of ambient air into the chamber. The temperature and relative humidity during the measurements were measured by HOBO MX1102A with mean values of 23.1±0.3 °C and 47.9±2.3%, respectively.

The tested PCPs were randomly selected at a grocery store in Germany, including a Nivea Men Fresh Active Spray (hereafter body spray), a Balea Handcream Cocos & Lotusbüte (hereafter hand lotion), a Nivea Fresh Flower roll-on Deodorant Antitranspirant (hereafter roll-on deodorant), an Issey Miyake L'Eau d'Issey Pour Homme perfume (hereafter perfume), and a Batiste Original Dry Shampoo hair spray (hereafter hair spray). In both primary emission and oxidation experiments, the application of the PCPs was simulated in the chamber by a volunteer, who wore nitrile gloves, a protection suit (Tychem 4000, DuPont, DE, USA), an FFP1 activated carbon face mask (3M, MN, USA) to minimize human-related VOC emissions and ozone-human surface reactions. The volunteer was present in the chamber only when applying the PCPs and left the chamber immediately after the application. In the primary emission experiments, two mixing fans were placed in two opposite corners in the chamber to increase mixing intensity of chamber air. The mixing fans were not used in the oxidation experiments since they resulted in significant aerosol wall loss rates and coagulation rates, which are unfavorable for characterizing new particle formation.

Before the experiment campaign, the chamber walls and floors were cleaned with de-ionized water and ethanol (70%) and then steam-cleaned with a Black+Decker Steamitt glove. The chamber was flushed with 100% filtered air at an air exchange rate (AER) of ~3 h^{-1} overnight before the day of the experiments. In the oxidation experiments, the ozone was added to the supply air duct by using an ozone generator (Jelight Company, Inc.). The ozone generator was fed with ultra-high purity oxygen at a constant rate. Ozone injection started at least 6 hours before the oxidation experiments to make sure the background VOC and particle concentrations reached a steady state or quasi-steady state. Before ozone injection, the background NCA and UPF concentrations were below 500 cm^{-3}. After injecting the ozone, the background NCA and UPF concentrations in the empty chamber were at ~5000 and ~8500 cm^{-3}, respectively, which could be attributable to the reactions between gas-phase ozone and unsaturated organic materials that ubiquitously exist on indoor surfaces.[1–4] The mean background aerosol particle size distributions (PSDs) without and with ozone injection, along with the mean PSDs in the oxidation experiments that are shown in Figure 1, were presented in Figure S5. Preliminary tests were conducted to examine the stability of background particles before PCP application in the oxidation experiments in the empty chamber (Figure S6).

During the experiments, the MiniWRAS was placed on a desk at the center of the chamber. The SMPS and nCNC were placed outside of the chamber and sampled the particles through two home-built core sampling probes[5,6] through a chamber wall. The length of the core sampling probes was ~1.1 m. Particle loss in the probes was corrected according to Fu et al.[6] The activation efficiency of the nCNC was calibrated by the manufacturer by using nickel-chromium particles. During the measurements, the sum of upscan and downscan time was 4 minutes. However, due to rapid particle formation in this study, the total aerosol number concentration might change dramatically within 5-10 minutes. Therefore, the nCNC might not perfectly pick up the rapid changes in NCA number concentration immediately after the use of the PCPs. The activation efficiency of the nCNC was calibrated by the manufacturer by using nickel-chromium particles, while the chemical composition of the NCAs in this study is expected to be organic. It is known that the activation efficiency for organic NCAs is lower than metal or sulfuric acid NCAs.[7,8] The actual size distributions might slightly deviate from the reported ones and we might underestimate the magnitude of NCA size distributions. The expectation-maximization method was used to convert the nCNC raw data to particle size distributions. The core algorithm follows Cai et al.[9]

and the inversion method is similar to Chan et al.[10] Our nCNC has shallow responses at sizes above 2.4 nm, partially because it is tuned to capture the smallest NCAs at ~1.2 nm. This likely prevents a good overlap in the size distribution at ~3 nm between the SMPS and the nCNC. We measured the mean air velocity at more than 30 locations in the chamber; and by combining this information with the dimensions of the surfaces in the chamber, we were able to estimate the size-resolved particle deposition rate coefficients to chamber surfaces following the method in Lai & Nazaroff (2000). Along with the real-time particle size distribution measurements, we have calculated the PM_1 surface deposition rate coefficients, and they are more than one order of magnitude lower than the air exchange rate of 0.65 h^{-1} (oxidation experiments). Therefore, we do not expect surface deposition to significantly affect the calculation of the aerosol formation factor.

In the experiments with the I-CIMS, the I-CIMS sampled the chamber air from the return air duct via ½" PFA tubing with a tubing length of ~2.1 m. The supply air entered the chamber from four floor diffusers and left the chamber via four ceiling grilles. In the experiments with the Vocus PTR, the Vocus PTR sampled the air directly from the chambers with a PFA sampling line protruding 45 cm into the chamber through a hole in the wall. The sampling line consists of a piece of ½" PFA tubing (145 cm) and a piece of ¼" PFA tubing (64 cm). The supply air entered the chamber from two ceiling diffusers and left the chamber via two ceiling grilles. An extra vacuum pump provided a carrier flow of ~9 L min^{-1} for the I-CIMS or Vocus PTR. The sampling location of the Vocus PTR was different from that of the I-CIMS, which potentially caused different sampling efficiencies for oxygenated volatile organic compounds (OVOCs).

The suitability of the I-CIMS and Vocus PTR has been discussed in previous literature.[11] The I-CIMS is sensitive to oxygenated organics, acids, peroxides, inorganic acids, and halogenated compounds. However, it is not sensitive to OVOCs with a form of $C_xH_yO_1$. On the other hand, due to the lack of calibration standards for the I-CIMS, only the signals (counts per second) of the detected compounds were reported instead of volumetric mixing ratios. When sampling with the I-CIMS, the sample air was drawn through a critical orifice at 2 standard liters per minute (slpm) into the Aim reactor, which was held at 50 mbar. Iodide ions were generated by passing a 250 standard cubic centimeters per minute (sccm) flow of ultrahigh purity (UHP) N_2 over a permeation tube filled with a mixture of methyl iodide and toluene and then through a vacuum ultraviolet (VUV) ion source into the Aim reactor. The toluene acted as a photon absorber and later ionized methyl iodide to generate iodide ions. A 10 sccm flow of acetonitrile was continuously introduced to the Aim reactor as a dopant to minimize the influence of humidity on instrument sensitivity. During the measurement campaign, the ratio of $I{\cdot}H_2O^-$ and $I{\cdot}C_2H_3N^-$ abundance to I^- abundance were below 0.1‰. The background signal of the I-CIMS was obtained every 2 hours by flowing UHP N_2 into the inlet for 1 minute. Data were recorded with a frequency of 1 Hz.

The Vocus PTR is highly sensitive to VOCs such as alkenes, aromatics, alcohols, aldehydes, ketones, acids, esters, ethers, and many other compounds. However, it cannot detect VOCs with a proton affinity lower than water, such as alkanes. The Vocus PTR sampled the air at a flow rate of 150 sccm. The background measurements were performed by flowing synthetic air into the inlet for at least 6 hours after the campaign. The instrument was calibrated before and after the campaign with a multi-VOC mixture that was dynamically diluted by synthetic air. The mixture contains methanol, acetonitrile, acetaldehyde, ethanol, acrylonitrile, acetone, dimethyl sulfide, isoprene, methyl vinyl ketone, methyl ethyl ketone, benzene, toluene, trimethylbenzene, α-pinene, xylene, caryophyllene, trichlorobenzene, siloxane D4, and siloxane D5. For the compounds without an authentic calibration standard, we assumed a reaction rate constant of 2.5×10^{-9} cm^3 s^{-1} for the proton transfer reactions of the parent compounds.[12] As mentioned in previous literature, the quantification of uncalibrated VOCs could result in uncertainties greater than 50%.[13,14] The Data were recorded with a temporal resolution of 4 seconds. The mass resolving power of the two instruments was ~10000 m dm^{-1}. Data analysis of the I-CIMS and Vocus PTR was performed using the Tofware package (v3.1.2) in Igor Pro environment (WaveMetrics, OR, USA).

The molecular formulas were assigned to the ions detected by the I-CIMS and Vocus PTR based on the mass-to-charge ratio and previous literature on indoor I-CIMS and PTR-MS measurements. Due to the relatively high collision energy in the drift tube of the Vocus PTR, fragmentation is expected for certain VOCs (e.g., aldehydes and alcohols). We were able to assign multiple detected ions to fragments based on previous literature on indoor PTR-MS measurements. However, sometimes it is difficult to determine the relative contributions of a fragment and a protonated parent ion to a detected ion when they share the same molecular formula, which is a known limitation when measuring the abundance of gas-phase VOCs solely by using a PTR-MS. In the indoor environment, this could happen to a part of the detected ions with a form of $C_xH_y^+$ and could lead to an overestimation of the abundance of hydrocarbons and an underestimation of OVOCs. For example, the detected ion $C_5H_9^+$ could be attributed to protonated isoprene, but also fragmentation of aldehydes, such as nonanal, octanal, decanal, and heptanal.[15] Therefore, in this study, we analyzed the data with caution and tried not to overinterpret the data.

Uncertainties Regarding Quantifying Ethanol with the Vocus PTR

Even though we estimated the mixing ratio of ethanol based on the sensitivity that was obtained from instrument calibration against the authentic calibration standards, the estimated mixing ratio may exhibit greater uncertainties compared to the heavier compounds in the multi-VOC mixture standard since the mass-to-charge ratio of ethanol is in the "suppressed range" of the Vocus PTR with relatively low transmission.[16,17] This may affect the calculation of emission factors (EFs) for the perfume, body spray, and hair spray, as ethanol is the predominant emitted VOC. However, we do not expect the uncertainty of estimating the ethanol mixing ratio to be greater than ±50%.

Details on Calculation of VOC Emission Factors (EFs)

Gas-phase VOC EFs for each tested PCP were estimated based on measurements obtained with the Vocus PTR during the primary emission experiments. The EF of a VOC represents the ratio of the emitted mass of the VOC to the mass consumption of the product of use (Equation S1). The emitted mass of a VOC is calculated as Equation S2, where, $VOC_i(t)$ is the concentration of an emitted VOC as a function of time, $VOC_{i,background}$ is the background concentration of VOC_i in the empty chamber before the experiment, Q is the volumetric airflow rate of the chamber, $t0$ is when the tested PCP is used, and $t1$ is when the concentration of the emitted VOC drops back to the background level. The assumption of using this method to calculate the EFs is that all the emitted VOCs from the PCPs can be flushed out of the chamber via ventilation when the VOC concentrations drop back to the background level. For the roll-on deodorant and hand lotion, the EFs only represent the first-hour emission factors.

$$EF_i(mg/g) = \frac{Mass\ of\ emitted\ VOC_i}{Mass\ consumption\ of\ product} \qquad \text{Equation (S1)}$$

$$Emitted\ mass\ of\ VOC_i(mg) = \int_{t0}^{t1} [VOC_i(t) - VOC_{i,background}]\ Q\ dt \qquad \text{Equation (S2)}$$

Indoor VOCs can partition between the gas phase and indoor surfaces by nature.[4,18] Upon the release of VOCs from the tested PCPs, they possibly partition onto indoor surfaces, which could lead to an underestimation of the EFs. To minimize this effect, the primary emission experiments were conducted at an outdoor AER of 3 h^{-1}, which is higher than the majority of the buildings in Europe. This is the largest outdoor AER that the ventilation system can achieve while keeping the chamber positively pressurized. Using a high AER can significantly reduce the relative fractions of emitted VOCs that partition onto indoor surfaces, further preventing underestimation of the EFs. We have estimated the first-order decay rates of VOC concentrations after the application of the PCPs for all the VOCs reported in the primary emission experiments in this study. The majority of the VOCs exhibit first-order decay rates of their concentrations slightly higher (~0-10%) than the room AER of 3 h^{-1}, indicating that the underestimation due to sorption onto indoor surfaces is expected to be less than 10%. An example is shown in Figure S7, which is the fitting of concentration decay of ethanol after using hair spray in the primary emission experiment. The fitted first-order decay rate of concentration is 3.24 h^{-1}.

Several VOCs of interest exhibited first-order decay rates lower than the room AER in the primary emission experiments. A potential reason is that these VOCs rapidly partition onto indoor surfaces and then gradually desorb from the surfaces to the gas phase. This effect can prolong the emission of PCP-related VOCs in indoor environments. An example is $C_8H_{10}O_2$ (tentatively phenoxyethanol), which was emitted from the hand lotion and exhibited a first-order decay rate of 1.42 h^{-1} in its concentration (Figure S8). The VOCs influenced by such an effect may have a relatively large molecular weight or lower saturation vapor pressure, with a carbon number greater than 10. The underestimation of EFs for these VOCs could potentially be greater than 10% due to surface sorption. However, since we assume that all the emitted VOCs from the PCPs can be flushed out of the chamber via ventilation and the method for calculating the mass of emitted VOCs requires integration over time till the VOC concentrations drop back to the background level, we would not expect the partitioning effect to significantly affect the EF calculation.

VOC Oxidation by Hydroxyl and Nitrate Radicals

In general, we expect ozone chemistry to be the dominant mechanism of oxidizing reactive VOCs that are released from the PCPs. This is partially supported by the agreements in the detected gas-phase oxidation products of monoterpenes with previous chamber studies.[19–21] However, we do realize that other radicals, such as hydroxyl (OH) and nitrate radicals (NO_3), could also contribute to VOC oxidation. Ozonolysis of alkene, such as monoterpenes, can produce OH. Multiple studies have estimated the OH yields from reactions between ozone and different alkenes.[22–24] Weschler and Shields have estimated the indoor OH production rates based on typical indoor ozone and alkene concentrations, which range from 9.5×10^{-8} to 2.6×10^{-4} ppb s^{-1}.[25] Despite the high reaction rate coefficients of OH, real-time OH measurements with laser-induced fluorescence–fluorescence assay by gas expansion (LIF-FAGE) techniques indicate that indoor OH did not exceed 1.5×10^{6} cm^{-3} (6.1×10^{-5} ppb) during ozonolysis of high concentration of indoor terpenes.[26] Given the ozone concentration in the chamber of 35 – 40 ppb, the OH radical concentration will be approximately 6 x 10^5 lower than the ozone concentration. Therefore, we do not expect indoor OH to contribute significantly to VOC oxidation in this study.

In the oxidation experiments shown in Figure S3, we have detected several nitrogen- (N-) containing oxidation products. Concentrations of NO and NO_2 were ~5-6 ppb in the empty chamber before ozone injection. This might be caused by unusually high concentrations of nitrogen oxides outdoors.Upon the injection of ozone, NO_2 concentration increased to ~8-9 ppb and NO concentration decreased to ~1-1.5 ppb. At the same time, the signal of N_2O_5 detected by the I-CIMS increased gradually, indicating the formation of NO_3. The N-containing HOMs might be formed by NO_3-induced oxidation. It is also possible that they were the organic nitrates formed from peroxy radicals (RO_2), which are produced by ozone-initiated auto-oxidation of alkenes, and NO, as NO was not depleted in the experiments. Due to the lack of calibration standards, we were not able to quantify the concentration of N_2O_5, therefore, could not estimate the NO_3 abundance. However, given the concentration ratio of ozone to NO_x (Table S5), we do not expect NO_3-initiated oxidation to contribute significantly.

Comparison with Atmospheric VOC Emission Inventories

A large portion of the VOCs emitted from the PCPs will end up in the atmosphere due to building ventilation, which contributes to atmospheric organic gases, ozone formation, and SOA formation. As the VOC emissions from transportation have decreased in developed countries, VOC emissions from PCPs to urban atmosphere have gained significant attention in recent years. Multiple recent studies compiled VOC emission inventories of PCPs. While we demonstrated that ethanol dominates in VOC emissions from the tested hair spray, body spray, and perfume, the VOC emission inventory compiled by McDonald et al. indicates that alcohols, ketones, and esters are the main contributors to VOC emissions from hair care and perfumes.[27] This inventory also reported total VOC emission factors for hair care, perfumes, and lotions to be 310±260, 490±270, and 540±290 mg g^{-1}, respectively. While decamethylcyclopentasiloxane (siloxane D5) is recognized as the tracer compound for PCPs in previous

VOC emission inventories[28–31], interestingly, we did not identify its emissions in the tested PCPs. Monoterpenes are reported to be the most abundant anthropogenic fragrance from volatile chemical products in these emission inventories. Similarly, we also found that monoterpenes exhibit the highest EFs in the fragrant VOCs emitted from the tested PCPs, except for the hand lotion.

Implications for Indoor Air Quality and Study Limitations

The study reveals that the reactive VOCs used as fragrant additives in PCPs can readily react with the common indoor gaseous oxidant, ozone, leading to the formation of gas-phase oxidized organic compounds and a substantial number of UFPs. These UFPs could effectively deposit in the pulmonary region in the human respiratory tract. Importantly, the study demonstrated that the reactive VOCs from the fragranced PCPs are not limited to the commonly reported monoterpenes, but also include monoterpene derivatives (e.g. linalyl acetate), sesquiterpenes, and other terpene-related fragrant chemicals (e.g. ionone and OTNE) possessing unsaturated C=C bonds, all of which undergo indoor oxidation as well. To mitigate potential indoor air pollution from the perspective of secondary chemistry of these fragrant compounds, building engineering approaches (e.g., activated carbon-based filters, catalytic decomposition, IAQ-based demand control) can be used to limit indoor oxidants, such as ozone. Alternative fragrant compounds with low reactivity, such as eucalyptol (a saturated compound), could be also considered, or chemical modifications could be implemented to reduce their reactivity.

The study highlights that indoor use of PCPs can significantly increase human inhalation exposure to VOCs and particles, as demonstrated through the simulated application of the PCPs without and with ozone respectively. However, the VOC emission dynamics in real-life scenarios may differ from those observed in simulated experiments. Differences in surface properties in realistic conditions, such as body temperature, partitioning between clothing and indoor air, and solubility on human skin, could influence the emission rate of VOCs from the human body to indoor air. For example, human skin has a temperature higher than the tested Kimwipe and different surface chemical properties, which may lead to a higher emission rate of VOCs in actual usage. When using the spray products, the impact of the sprayed ingredients and the partition of gas-phase compounds on the human body may weaken the initial burst emission but also prolong the emission into indoor air later on. This delayed emission would then result in a personal cloud of slowly desorbing VOCs[32], the underlying intention behind the use of fragranced PCPs. The reactive VOCs in the personal cloud could further react with indoor oxidants (O_3, OH, and NO_3)[33,34], resulting in localized exposure to elevated levels of oxidation products. However, the health information is currently not available for such substances.[35,36] There exists a large set of reaction products with unevaluated toxicities and knowledge of this underlying chemistry and its products can guide us towards selecting compounds for which toxicity data should be generated. Since these products are generated in the vicinity of the breathing zone, it makes them all the more important.

Given the AER of 0.65 h^{-1}, the ozone concentrations in the oxidation experiments were higher than in most realistic indoor environments.[37] We would expect lower formation rates of HOMs and aerosols in reality due to expected lower ozone levels at this AER. On the other hand, if the ventilation and ozone conditions remain the same, using PCPs in a real indoor environment may result in a greater particle concentration than in the test chamber due to human-emitted ammonia. The experiments in this study were conducted in an unoccupied environment, however, in reality, human-emitted ammonia from the skin and breath could lead to high gas-phase ammonia concentrations[38], which facilitates the formation of ammonium salts from the reaction between organic acids, which are generated from the ozonolysis of alkenes, and human-emitted ammonia. This effect could result in higher particle formation rates.

We tested five products in this study to provide novel insight into the impact of using PCPs on the chemical compositions of indoor air. However, one clarification is that the results for each tested PCP should not be generalized for other products in that category. We do acknowledge that the variability in products, even in the same category, can significantly affect the EFs and aerosol formation potential. This pioneering study aims to map out the scale of chemical impacts indoors rather than providing a

comprehensive assessment of all products available. On the other hand, many environmental factors can influence the fate and oxidation of the VOCs emitted from the PCPs indoors, such as outdoor ozone and nitrogen oxide levels, building air exchange rate, ventilation control strategy, condensation sink due to existing particles and indoor surfaces, daylighting and indoor lighting, and surface partitioning. In addition, pulse VOC emissions upon the use of PCPs may cause rapid VOC partitioning from the gas phase to indoor surfaces. The surface-bound reactive VOCs can react with ozone heterogeneously and contribute to ozone losses on indoor surfaces.

Table S1. The assignment of several VOCs from the TD-GC-MS analysis.

Ions detected by Vocus PTR	Identification by TD-GC-MS
C2H6OH+	Ethanol
C3H8O2H+	Propylene Glycol
C10H14H+	o-Cymene
C6H14O3H+	Dipropylene glycol
C10H16H+	α-Pinene
	β-Pinene
	β-Myrcene
	Limonene
	γ-Terpinene
C10H18OH+	Eucalyptol
	Linalool
C10H20OH+	Dihydro-α-terpineol
	Citronellol
	Dihydromyrcenol
C12H20O2H+	Linalyl acetate
	α-Terpinyl acetate
C10H22OH+	1,1-Dimethoxyoctane
C9H18OH+, C9H17+	Nonanal

Table S2. Summary of recommended rate coefficients for reactions of O_3 with fragrance compounds from the PCPs.

**Compounds**	k_{298} (cm^3 $molec.^{-1}$ s^{-1})
α-pinene[39]	9.6×10^{-17}
β-pinene[39]	1.9×10^{-17}
limonene[39]	2.2×10^{-16}
β-myrcene[39]	4.7×10^{-16}
γ-terpinene[39]	1.6×10^{-16}
eucalyptol	Not found
linalool[40]	4.1×10^{-16}
citronellol[41]	2.4×10^{-16}
dihydro-α-terpineol	Not found
α-terpinyl acetate	Not found
linalyl acetate	Not found
*ionone[42]	1.9×10^{-17}
*α-isomethyl ionone	Not found

*tetramethyl acetyloctahydronaphthalenes (OTNE)[43]	2.1×10^{-18}
*acetyl cedrene[43]	$<2.2\times10^{-18}$

*The compounds were assigned empirically based on the formulas measured by the Vocus PTR, and not confirmed by the TD-GC-MS analysis.

Table S3. The ingredient chart of PCPs by the manufacturer. The bold font denotes that the compound has been detected with the Vocus PTR or I-CIMS in the primary emission experiments.

Roll-on deodorant	***Molecular Formulas***
Water	
Isoceteth-20	
Paraffinum Liquidum	
**Butylene Glycol**	**C4H10O2**
Glyceryl Isostearate	
Glycerin	C3H8O3
Parfum	
Polyquaternium-16	
Maris Limus Extract	
Ostrea Shell Extract	
Persea Gratissima Oil	
**Propylene Glycol**	**C3H8O2**
PEG-150 Distearate	
**Phenoxyethanol**	**C8H10O2**
**Linalool**	**C10H18O**
**Benzyl Alcohol**	**C7H8O**
**Limonene**	**C10H16**
Hand lotion	***Molecular Formulas***
Water	
Glycerin	C3H8O3
Cetearyl Alcohol	
Isopropyl Palmitate	C19H38O2
**Dicaprylyl Ether**	**C16H34O**
Glyceryl Stearate	C21H42O4
Nelumbo Nucifera Flower Extract	
Cocos Nucifera Oil	
Parfum	
Sodium Hydroxide	
Potassium Sorbate	
**Phenoxyethanol**	**C8H10O2**
Sodium Cetearyl Sulfate	
Sodium Benzoate	
Lactic Acid	C3H6O3
Tocopherol	
Glycine Soja Oil	

Hair Spray	***Molecular Formulas***
Butane	C4H10
Isobutane	C4H10
Propane	C3H8
Oryza Sativa Starch	
**Alcohol Denat**	**C2H6O**
Parfum	
**Limonene**	**C10H16**
**Linalool**	**C10H18O**
**Geraniol**	**C10H18O**
Benzyl Benzoate	C14H12O2
Distearyldimonium Chloride	C38H80ClN
Cetrimonium Chloride	C19H42ClN
Perfume	***Molecular Formulas***
**Alcohol**	**C2H6O**
Parfum	
Water	
**Dipropylene Glycol**	**C6H14O3**
Butylphenyl Methylpropional	C14H20O
**Limonene**	**C10H16**
**Linalool**	**C10H18O**
Ethylhexyl Methoxycinnamate	C18H26O3
Butyl Methoxydibenzoylmethane	C20H22O3
**Eugenol**	**C10H12O2**
**Citronellol**	**C10H20O**
**Geraniol**	**C10H18O**
**Citral**	**C10H16O**
**Coumarin**	**C9H6O2**
**Hydroxycitronellal**	**C10H20O2**
**Farnesol**	**C15H26O**
**Cinnamal**	**C9H8O**
**Isoeugenol**	**C10H12O2**
**Butylated Hydroxytoluene**	**C15H24O**
Body Spray	***Molecular Formulas***
Butane	C4H10
**Alcohol Denat**	**C2H6O**
Isobutane	C4H10
Propane	C3H8
Parfum	
Octenidine HCl	
**Ethylhexylglycerin**	**C11H24O3**
Maris Limus Extract	
Ostrea Shell Extract	

Persea Gratissima Oil	
**Octyldodecanol**	**C20H42O**
**Propylene Glycol**	**C3H8O2**
Water	
Sodium Benzoate	
Potassium Sorbate	
Lactic Acid	C3H6O3
Benzophenone	C13H10O
**Linalool**	**C10H18O**
**Limonene**	**C10H16**
**Geraniol**	**C10H18O**
**Citronellol**	**C10H20O**
**Alpha-Isomethyl Ionone**	**C14H22O**
**Eugenol**	**C10H12O2**
**Citral**	**C10H16O**

Table S4. The particle growth rates[44] in four new particle formation events (Figure 1) due to the use of PCPs with the presence of indoor ozone, along with the comparisons with those reported in urban and remote sites.

Size range (nm)	Growth rate (nm h^{-1})				
	Roll-on deodorant	Hand lotion	Hair spray	Perfume	Body spray
3-7	--	28	29	83.7	86.8
7-20	--	36	18.4	92	61.7
Urban Sites	Beijing[45]		Beijing[46]	Shanghai[47]	Shanghai[48]
3-7	2.7		3.5 (0.5-9)	6.9 (2.7-14.47)	10.9±9.8
7-20	5.5		--	10.88 (8.16-18.58)	11.4±9.7
Remove Sites	Jungfraujoch[49]	Hyytiala[50]	Hyytiala[51]	Chacaltaya[52]	
3-7	5.3±3.5	3.6	2.5±1.9	6.86	
7-20	5.7±2.2	4.2		7.62	

Table S5. The emission factor for the VOCs that are included in Figure 2 from the primary emission experiments.

m/z from Vocus PTR	Assigned Formulas	Tentative assignment	Emission Factor (μg g^{-1})				
			Roll-on deodorant	Hand lotion	Hair Spray	Perfume	Body spray
45.033	C2H5O+	Acetaldehyde + fragments	2219	116	182	6117	858
47.013	CH3O2+	Formic acid	0	0	77	1139	380
47.049	C2H7O+	Ethanol	0	0	61737	812613	299402
57.033	C3H4OH+		111	2	153	1312	280
59.049	C3H7O+	Acetone + propanal	0	0	126	8053	1210

69.070	C5H9+	Isoprene + fragments	744	20	48	1965	207
71.013	C3H2O2H+		60	1	29	15	37
71.086	C5H11+	Pentanol fragment + isomers	322	6	33	532	78
73.028	C3H4O2H+		655	0	15	0	12
75.044	C3H6O2H+		21	0	7	1863	86
77.060	C3H9O2+	Propylene glycol	28	0	9	804	242
79.054	C6H7+	Benzene + fragments	459	11	69	1931	245
83.086	C6H11+	Hexanal fragment + isomers	406	29	12	1272	237
85.101	C6H13+		231	6	24	121	27
87.044	C4H6O2H+		324	7	8	1332	51
91.054	C7H7+		746	105	120	401	16
91.075	C4H10O2H+		1510	4	2	32	4
95.049	C6H6OH+		323	139	0	1286	53
95.086	C7H11+		130	5	41	1296	199
97.101	C7H13+	Heptanal fragment + isomers	159	3	6	3760	89
99.080	C6H10OH+		224	3	2	2351	238
101.060	C5H8O2H+		207	2	6	587	31
103.039	C4H6O3H+		257	2	5	379	17
103.075	C5H10O2H+		98	4	2	65	2
105.055	C4H8O3H+		59	0	0	968	42
105.070	C8H8H+		70	7	47	520	13
107.086	C8H11+		197	2	4	171	22
111.117	C8H15+		127	2	12	843	17
114.091	C6H11NOH+		122	8	34	603	157
115.075	C6H10O2H+		115	2	2	4201	149
117.055	C5H8O3H+		60	1	4	454	81
119.086	C9H11+		106	5	3	111	21
121.101	C9H13+		138	2	5	131	29
127.148	C9H18H+		30	1	45	0	33
129.127	C8H16OH+		79	1	35	57	4
131.070	C6H10O3H+		47	14	7	1326	49
131.086	C10H10H+		29	1	4	2011	44
131.107	C7H14O2H+		193	14	7	112	5
133.086	C6H12O3H+		32	1	1	1285	46
133.101	C10H12H+		87	2	37	152	19
135.102	C6H15O3	Dipropylene glycol	536	12	8	27347	1337
137.060	C8H8O2H+		221	3	13	113	36
137.132	C10H17+	Monoterpenes	1241	148	1204	32220	4953
139.075	C8H10O2H+		666	1071	0	3183	14
139.148	C10H18H+		180	12	5	108	170

141.055	C7H8O3H+		12	5	6	98	8
141.164	C10H21+		134	0	14	0	8
143.107	C8H14O2H+		114	4	14	225	20
145.122	C8H16O2H+		500	26	14	214	16
153.127	C10H16OH+		101	8	25	1021	110
153.164	C11H21+		0	0	0	3022	0
155.143	C10H19O+	Eucalyptol + linalool	104	4	27	891	113
157.122	C9H16O2H+		114	4	8	182	11
157.159	C10H21O+	Dihydro-α-terpineol + isomers	513	3	11	467	84
165.091	C10H12O2H +		103	3	0	794	89
171.138	C10H18O2H +		58	11	6	87	11
171.174	C11H22OH+		202	0	1	45	0
173.132	C13H16H+		29	3	13	0	40
185.190	C12H24OH+		246	0	0	94	0
187.133	C10H18O3H +		69	1	8	232	123
187.169	C11H22O2H +	3,5,5-Trimethylhexy l acetate	41	0	68	96	5
193.159	C13H20OH+		83	2	0	100	187
195.174	C13H22OH+		74	0	0	2193	26
201.185	C12H24O2H +		11	1	18	97	0
205.195	C15H24H+	Sesquiterpenes	205	2	10	3617	1265
207.174	C14H22OH+		0	0	19	354	203
225.185	C14H24O2H +		0	0	0	1599	128
235.206	C16H26OH+		207	2	3	4395	1223
243.268	C16H34OH+		36	29	0	0	0
247.206	C17H26OH+		29	0	1	3162	401
259.206	C18H26OH+		0	0	0	1435	0

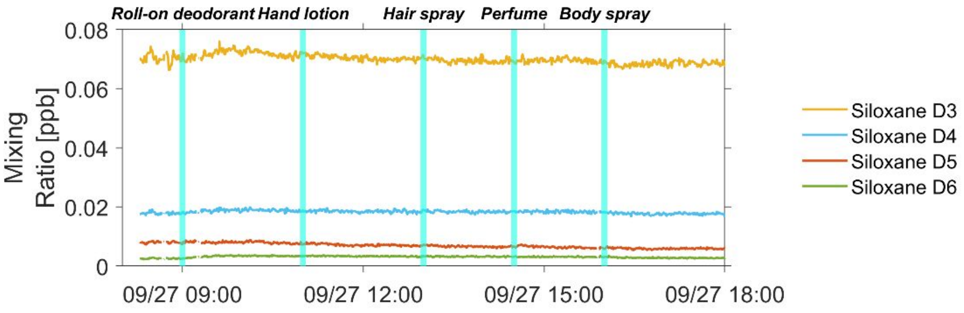

Figure S1. Concentrations of cyclic methyl siloxanes measured with the Vocus PTR in a primary emission experiment.

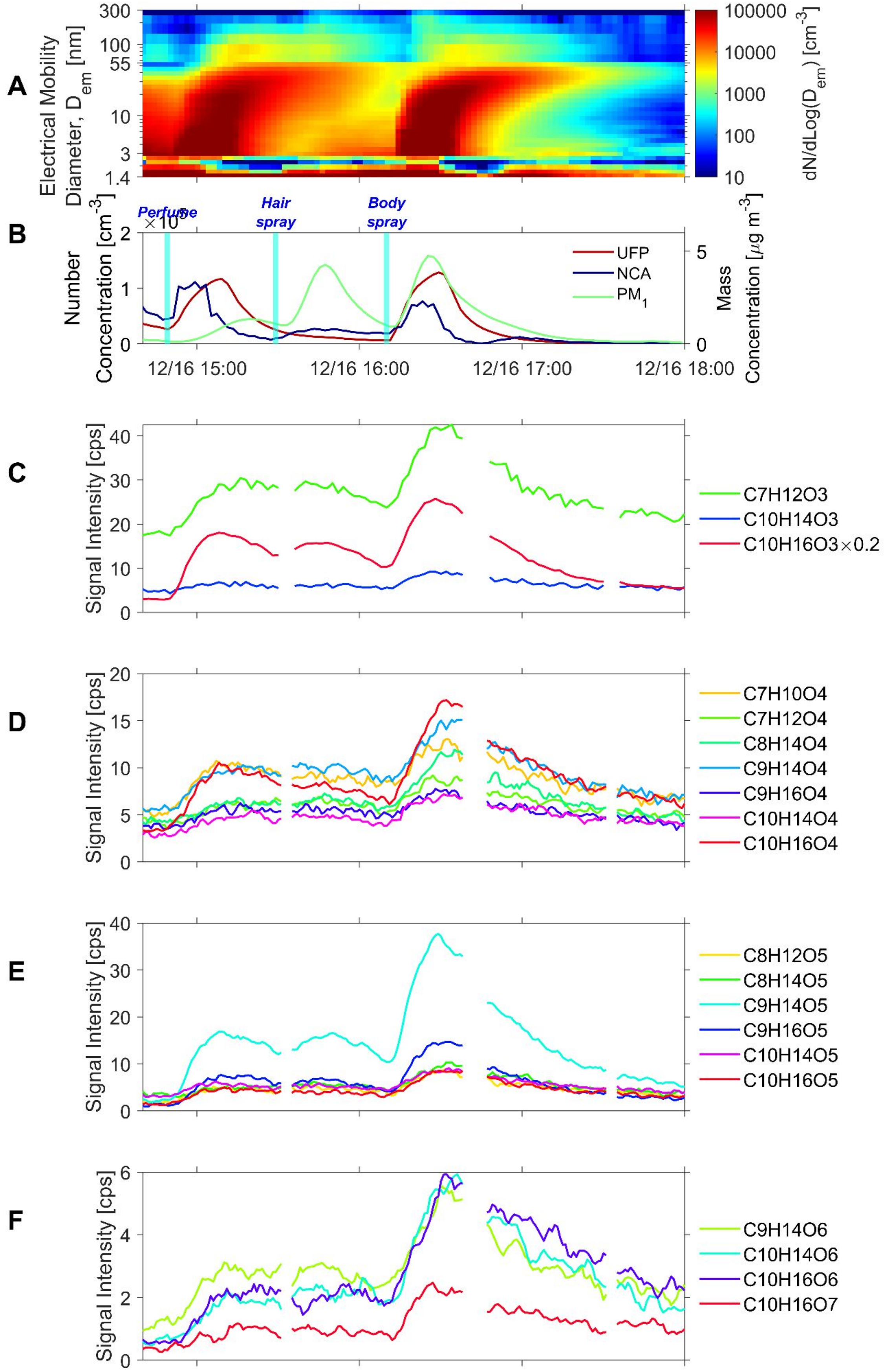
A
Electrical Mobility Diameter, D_{em} [nm]
$dN/dLog(D_{em})$ [cm^{-3}]
B
Perfume
Hair spray
Body spray
Number Concentration [cm^{-3}]
Mass Concentration [$\mu g\ m^{-3}$]
UFP
NCA
PM_1
12/16 15:00
12/16 16:00
12/16 17:00
12/16 18:00
C
Signal Intensity [cps]
C7H12O3
C10H14O3
C10H16O3×0.2
D
C7H10O4
C7H12O4
C8H14O4
C9H14O4
C9H16O4
C10H14O4
C10H16O4
E
C8H12O5
C8H14O5
C9H14O5
C9H16O5
C10H14O5
C10H16O5
F
C9H14O6
C10H14O6
C10H16O6
C10H16O7

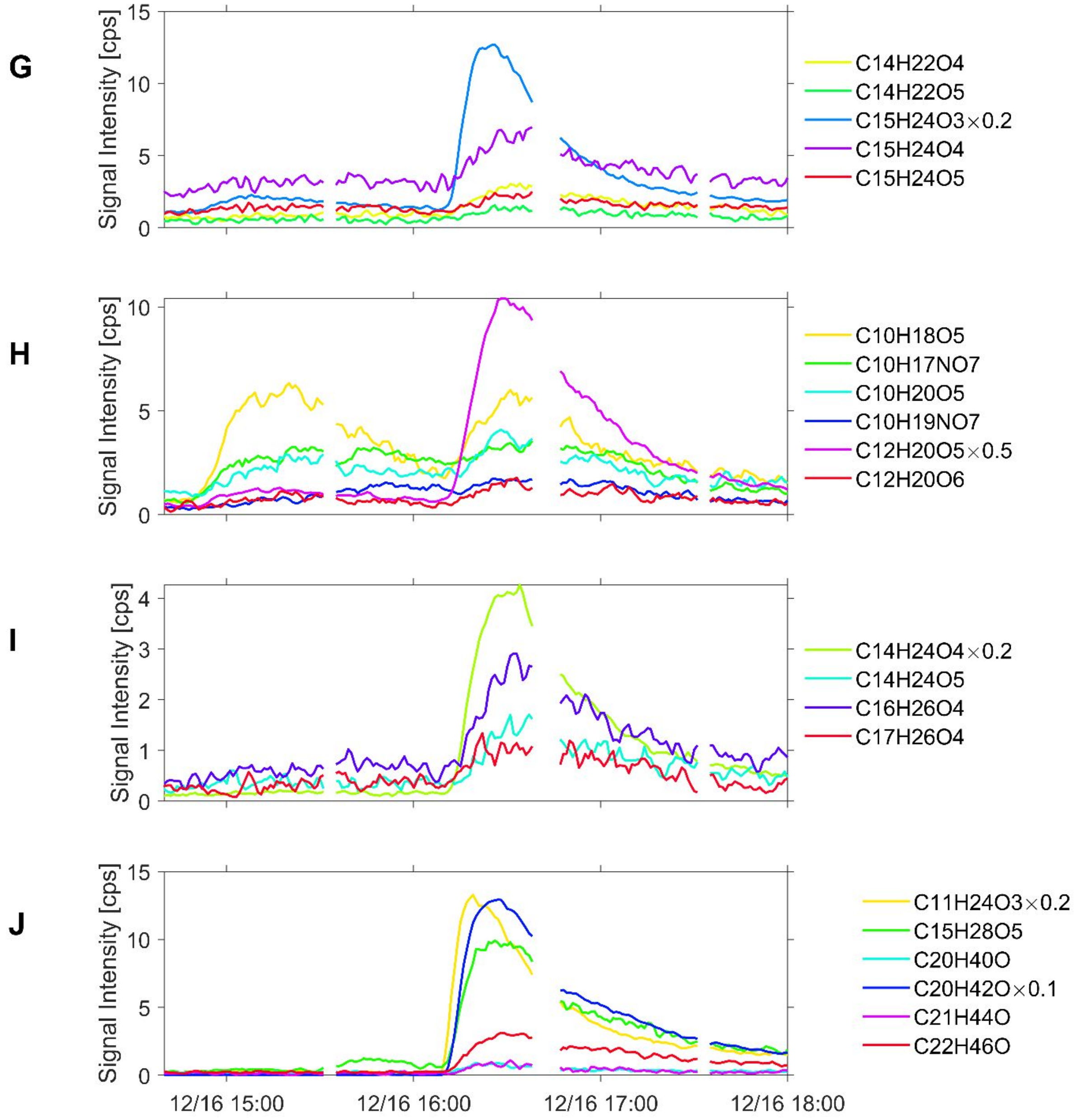

Figure S2A-J. (A) Time-series plot of aerosol number size distribution (dN/dLog(D_{em})); (B) concentrations of nanocluster aerosol (NCA; blue line), ultrafine particle (UFP; red line), PM_1 mass concentration (green line); (C-F) signal intensity of gas-phase oxidation products of monoterpenes with 3-7 oxygen atoms; (G) signal intensity of gas-phase oxidation products of sesquiterpenes; (H-I) signal intensity of gas-phase oxidation products of other terpene derivatives; and (J) directly emitted gas-phase organic compounds measured by the I-CIMS in a supplement **oxidation** experiment. The consumption of the perfume, hair spray, and body spray was 0.272, 2.85, and 1.49 g, respectively. This experiment was conducted with an AER of **1.83** h^{-1}, an ozone level of 26-30 ppb, and a NO_x level of 4.2-14.9 ppb. When injecting ozone before the oxidation experiment, we observed the formation of N_2O_5, which indicates the existence of nitrate radicals in the chamber. We have observed some compounds that were previously reported as the oxidation products from nitrate-radical-induced autooxidation, such as $C_{10}H_{17}NO_7$ and $C_{10}H_{19}NO_7$.

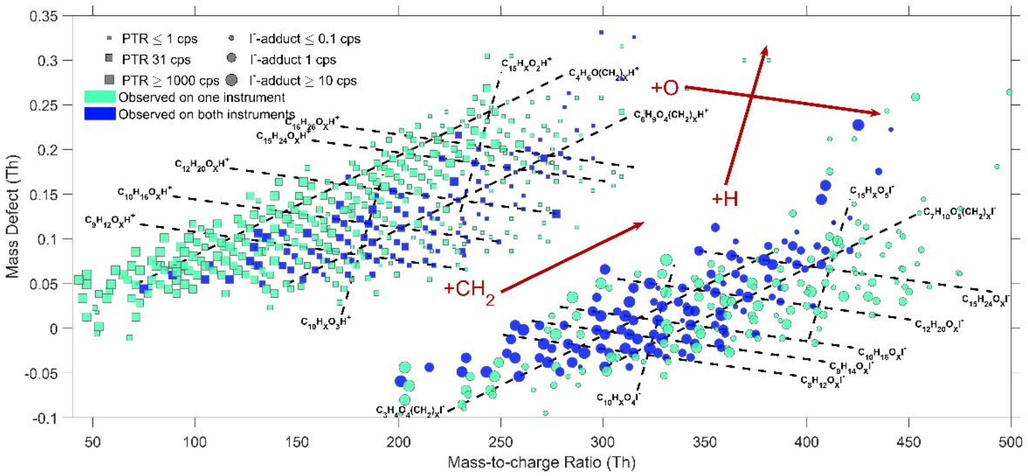

Figure S3. Mass defect plot of the gas-phase organic compounds detected by the I-CIMS and Vocus PTR in the oxidation experiments. The size of the marker represents the average raw signal intensity (counts per second, cps) in all oxidation experiments. The dashed lines and arrows indicate the patterns in their molecular formulas.

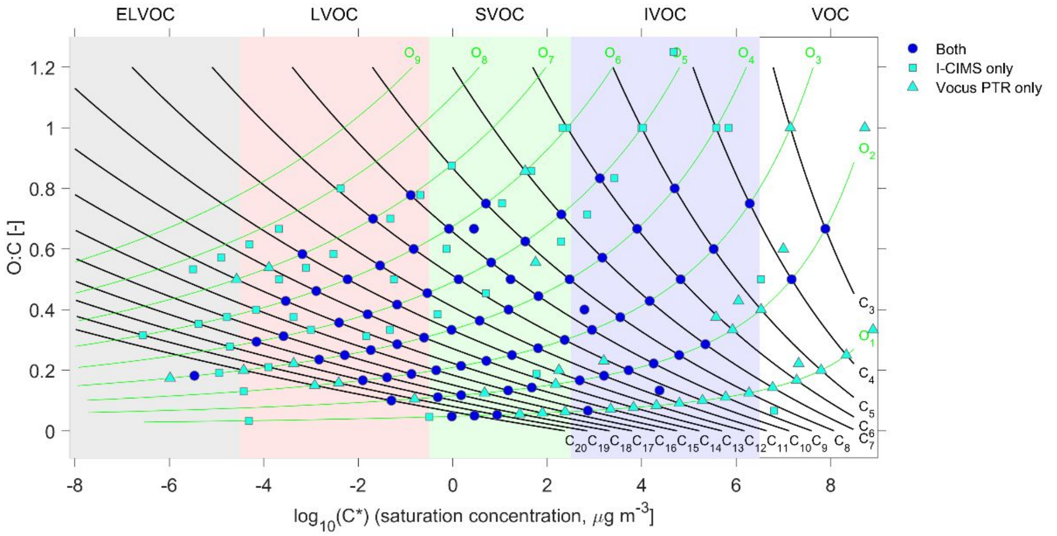

Figure S4. The distribution of the volatility of the oxidized organic compounds in the oxidation experiments of perfume, hair spray, and body spray, measured by the I-CIMS and Vocus PTR. The horizontal axis is the volatility at 300 K estimated following the method in Mohr et al.[53]

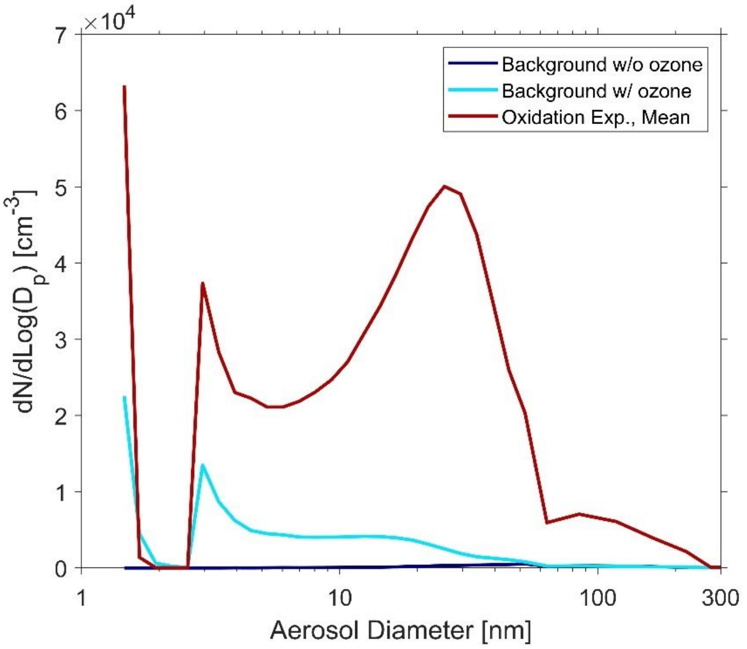

Figure S5. The mean aerosol particle size distributions (PSDs) in the test chamber during the background measurements with (light blue) and without (dark blue) the injection of ozone, along with the mean PSDs in the oxidation experiments shown in Figure 1 (red).

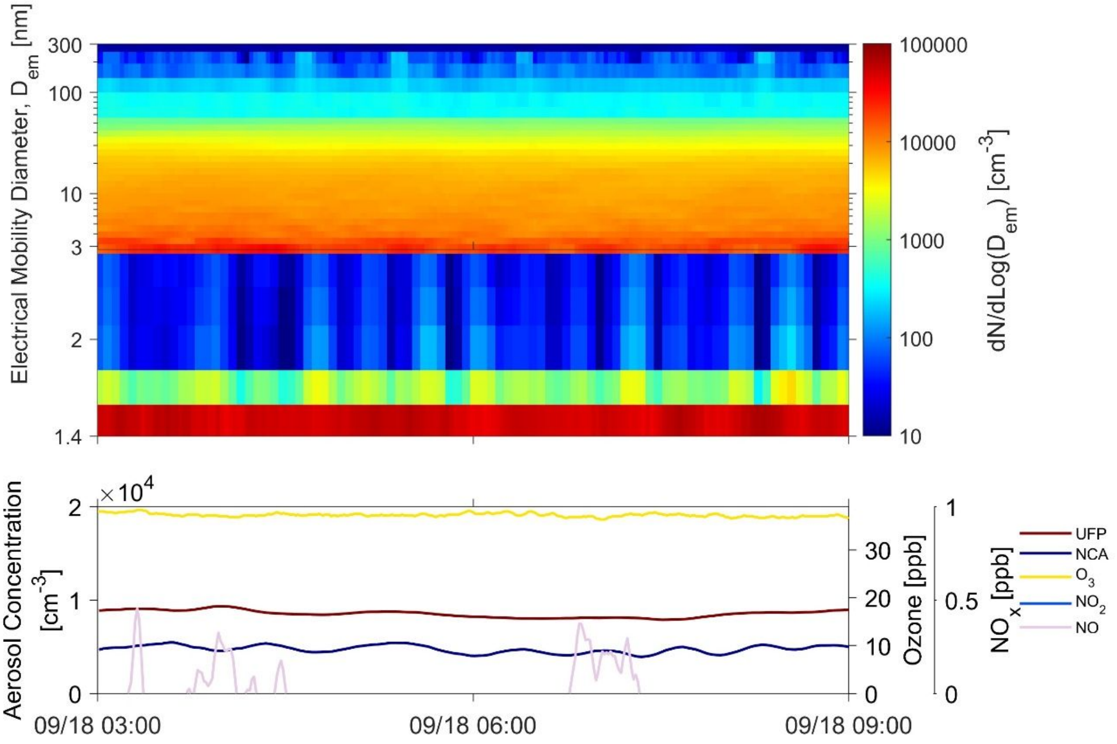

Figure S6. Background measurements of ozone, nitrogen oxides, and aerosol particles in the preliminary tests to examine the stability of background conditions.

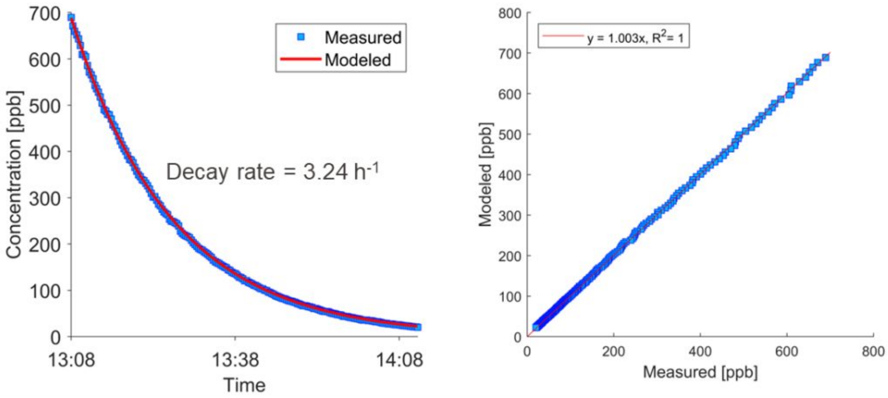

Figure S7. Fitting of the concentration decay for ethanol after the application of the hair spray in the primary emission experiment. The left plot shows the measured concentration and the modeled concentration with the fitted first-order decay rate. The right plot shows the quality of fitting.

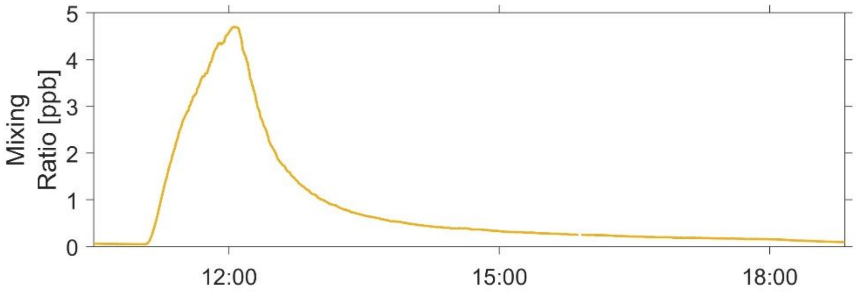

Figure S8. Concentration profile of $C_8H_{10}O_2$ (tentatively phenoxyethanol) in the primary emission experiment of the hand lotion. Note that the glass plate with the hand lotion was placed in the test chamber for one hour.

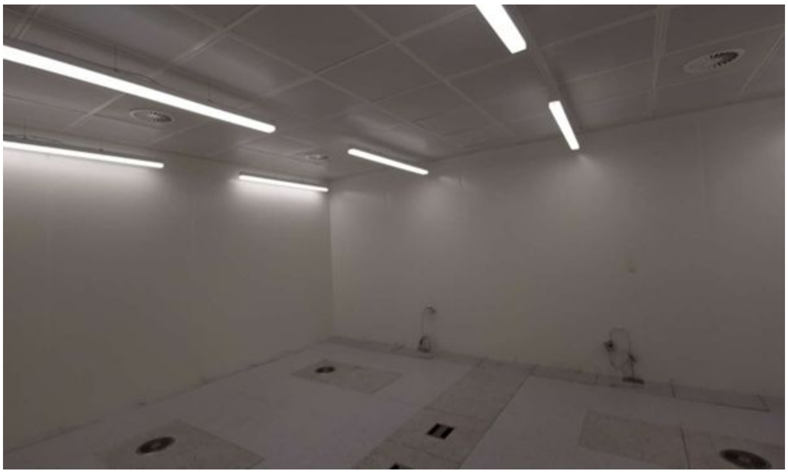

Figure S9. A picture of the empty chamber when it was being prepared for the experiments.

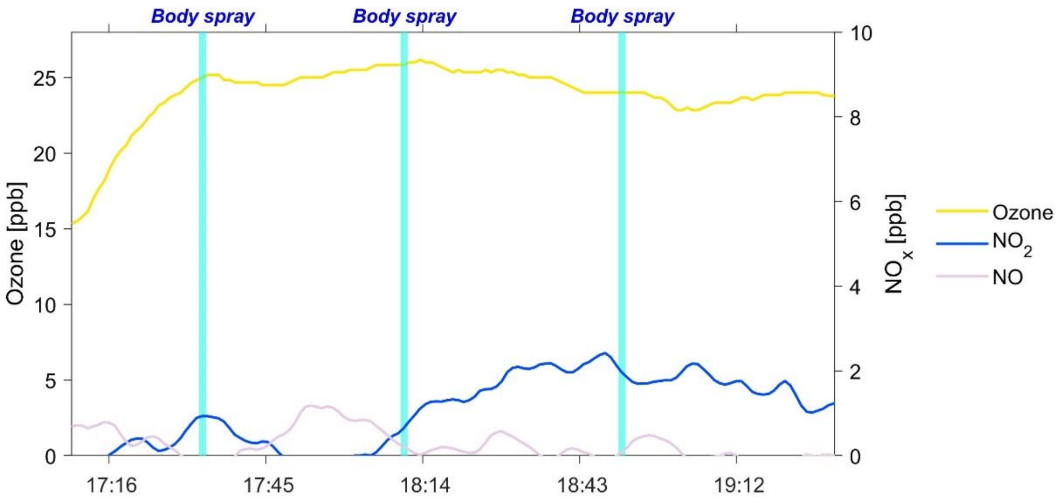

Figure S10. Preliminary test to examine the consumption of ozone by VOCs released from the PCPs. We found that ozone concentrations were stable when applying PCPs in the chamber. Using PCPs in the oxidation experiments would not significantly reduce ozone concentrations.

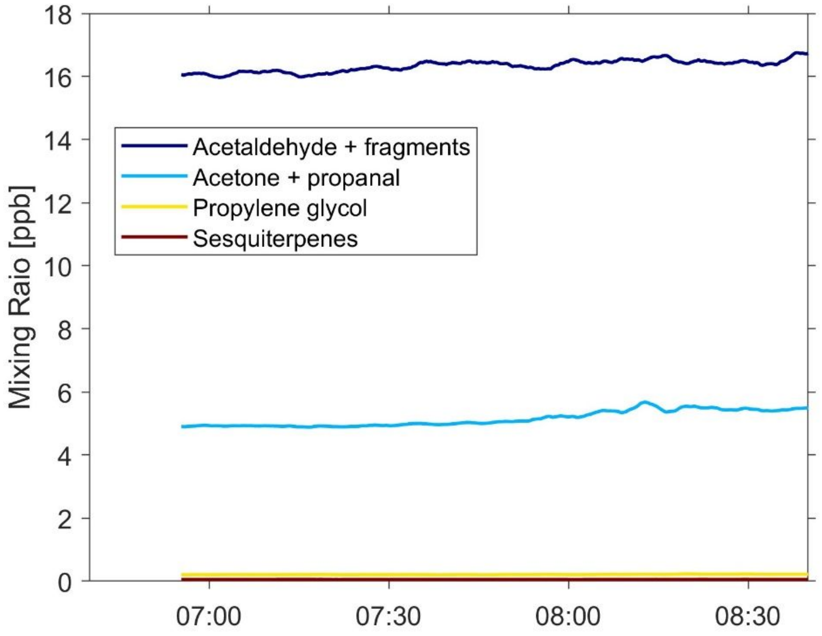

Figure S11. The time-series plot of several VOCs before the oxidation experiments that are shown in Figure 1. VOC concentrations were stable under the background conditions.

References

(1) Algrim, L. B.; Pagonis, D.; de Gouw, J. A.; Jimenez, J. L.; Ziemann, P. J. Measurements and Modeling of Absorptive Partitioning of Volatile Organic Compounds to Painted Surfaces. *Indoor Air* **2020**, *30* (4), 745–756.

(2) Weschler, C. J.; Nazaroff, W. W. Growth of Organic Films on Indoor Surfaces. *Indoor Air* **2017**, *27* (6), 1101–1112. https://doi.org/10.1111/ina.12396.

(3) Eichler, C. M. A.; Cao, J.; Isaacman-VanWertz, G.; Little, J. C. Modeling the Formation and Growth of Organic Films on Indoor Surfaces. *Indoor Air* **2019**, *29* (1), 17–29.

(4) Abbatt, J. P. D.; Morrison, G. C.; Grassian, V. H.; Shiraiwa, M.; Weschler, C. J.; Ziemann, P. J. How Should We Define an Indoor Surface? **2022**.

(5) Kangasluoma, J.; Franchin, A.; Duplissy, J.; Ahonen, L.; Korhonen, F.; Attoui, M. Operation of the Airmodus A11 Nano Condensation Nucleus Counter at Various Inlet Pressures and Various Operation Temperatures , and Design of a New Inlet System. **2016**, 2977–2988. https://doi.org/10.5194/amt-9-2977-2016.

(6) Fu, Y.; Xue, M.; Cai, R.; Kangasluoma, J.; Jiang, J. Theoretical and Experimental Analysis of the Core Sampling Method: Reducing Diffusional Losses in Aerosol Sampling Line. *Aerosol Science and Technology* **2019**, *53* (7), 793–801.

(7) Kangasluoma, J.; Kuang, C.; Wimmer, D.; Rissanen, M. P.; Lehtipalo, K.; Ehn, M.; Worsnop, D. R.; Wang, J.; Kulmala, M.; Petäjä, T. Sub-3 Nm Particle Size and Composition Dependent Response of a Nano-CPC Battery. *Atmos Meas Tech* **2014**, *7* (3), 689–700.

(8) Kangasluoma, J.; Kontkanen, J. On the Sources of Uncertainty in the Sub-3 Nm Particle Concentration Measurement. *J Aerosol Sci* **2017**, *112*, 34–51.

(9) Cai, R.; Yang, D.; Ahonen, L. R.; Shi, L.; Korhonen, F.; Ma, Y.; Hao, J. Data Inversion Methods to Determine Sub-3 Nm Aerosol Size Distributions Using the Particle Size Magnifier Data Inversion Methods to Determine Sub-3 Nm Aerosol Size Distributions Using the Particle Size Magnifier. **2018**, *11* (7), 4477–4491.

(10) Chan, T.; Cai, R.; Ahonen, L. R.; Liu, Y.; Zhou, Y.; Vanhanen, J.; Dada, L.; Chao, Y.; Liu, Y.; Wang, L. Assessment of Particle Size Magnifier Inversion Methods to Obtain the Particle Size Distribution from Atmospheric Measurements. *Atmos Meas Tech* **2020**, *13* (9), 4885–4898.

(11) Riva, M.; Rantala, P.; Krechmer, J. E.; Peräkylä, O.; Zhang, Y.; Heikkinen, L.; Garmash, O.; Yan, C.; Kulmala, M.; Worsnop, D. Evaluating the Performance of Five Different Chemical Ionization Techniques for Detecting Gaseous Oxygenated Organic Species. *Atmos Meas Tech* **2019**, *12* (4), 2403–2421.

(12) Liu, Y.; Misztal, P. K.; Xiong, J.; Tian, Y.; Arata, C.; Weber, R. J.; Nazaroff, W. W.; Goldstein, A. H. Characterizing Sources and Emissions of Volatile Organic Compounds in a Northern California Residence Using Space-and Time-resolved Measurements. *Indoor Air* **2019**, *29* (4), 630–644.

(13) Wang, N.; Ernle, L.; Beko, G.; Wargocki, P.; Williams, J. Emission Rates of Volatile Organic Compounds from Humans. *Environ Sci Technol* **2022**, *56* (8), 4838–4848.

(14) Tang, X.; Misztal, P. K.; Nazaroff, W. W.; Goldstein, A. H. Volatile Organic Compound Emissions from Humans Indoors. *Environ Sci Technol* **2016**, *50* (23), 12686–12694.

(15) Ernle, L.; Wang, N.; Bekö, G.; Morrison, G.; Wargocki, P.; Weschler, C. J.; Williams, J. Assessment of Aldehyde Contributions to PTR-MS m/z 69.07 in Indoor Air Measurements. *Environmental Science: Atmospheres* **2023**, *3* (9), 1286–1295.

(16) Holzinger, R.; Acton, W. J. F.; Bloss, W. J.; Breitenlechner, M.; Crilley, L. R.; Dusanter, S.; Gonin, M.; Gros, V.; Keutsch, F. N.; Kiendler-Scharr, A. Validity and Limitations of Simple Reaction Kinetics to Calculate Concentrations of Organic Compounds from Ion Counts in PTR-MS. *Atmos Meas Tech* **2019**, *12* (11), 6193–6208.

(17) Krechmer, J.; Lopez-Hilfiker, F.; Koss, A.; Hutterli, M.; Stoermer, C.; Deming, B.; Kimmel, J.; Warneke, C.; Holzinger, R.; Jayne, J. Evaluation of a New Reagent-Ion Source and Focusing Ion–Molecule Reactor for Use in Proton-Transfer-Reaction Mass Spectrometry. *Anal Chem* **2018**, *90* (20), 12011–12018.

(18) Wang, C.; Collins, D. B.; Arata, C.; Goldstein, A. H.; Mattila, J. M.; Farmer, D. K.; Ampollini, L.; DeCarlo, P. F.; Novoselac, A.; Vance, M. E. Surface Reservoirs Dominate Dynamic Gas-Surface Partitioning of Many Indoor Air Constituents. *Sci Adv* **2020**, *6* (8), eaay8973.

(19) Yu, J.; Cocker, D. R.; Griffin, R. J.; Flagan, R. C.; Seinfeld, J. H. Gas-Phase Ozone Oxidation of Monoterpenes: Gaseous and Particulate Products. *J Atmos Chem* **1999**, *34*, 207–258.

(20) Hammes, J.; Lutz, A.; Mentel, T.; Faxon, C.; Hallquist, M. Carboxylic Acids from Limonene Oxidation by Ozone and Hydroxyl Radicals: Insights into Mechanisms Derived Using a FIGAERO-CIMS. *Atmos Chem Phys* **2019**, *19* (20), 13037–13052.

(21) Zhao, J.; Häkkinen, E.; Graeffe, F.; Krechmer, J. E.; Canagaratna, M. R.; Worsnop, D. R.; Kangasluoma, J.; Ehn, M. A Combined Gas-and Particle-Phase Analysis of Highly Oxygenated Organic Molecules (HOMs) from α-Pinene Ozonolysis. *Atmos Chem Phys* **2023**, *23* (6), 3707–3730.

(22) Siese, M.; Becker, K. H.; Brockmann, K. J.; Geiger, H.; Hofzumahaus, A.; Holland, F.; Mihelcic, D.; Wirtz, K. Direct Measurement of OH Radicals from Ozonolysis of Selected Alkenes: A EUPHORE Simulation Chamber Study. *Environ Sci Technol* **2001**, *35* (23), 4660–4667.

(23) Kroll, J. H.; Donahue, N. M.; Cee, V. J.; Demerjian, K. L.; Anderson, J. G. Gas-Phase Ozonolysis of Alkenes: Formation of OH from Anti Carbonyl Oxides. *J Am Chem Soc* **2002**, *124* (29), 8518–8519.

(24) Rickard, A. R.; Johnson, D.; McGill, C. D.; Marston, G. OH Yields in the Gas-Phase Reactions of Ozone with Alkenes. *J Phys Chem A* **1999**, *103* (38), 7656–7664.

(25) Weschler, C. J.; Shields, H. C. Production of the Hydroxyl Radical in Indoor Air. *Environ Sci Technol* **1996**, *30* (11), 3250–3258.

(26) Rosales, C. M. F.; Jiang, J.; Lahib, A.; Bottorff, B. P.; Reidy, E. K.; Kumar, V.; Tasoglou, A.; Huber, H.; Dusanter, S.; Tomas, A. Chemistry and Human Exposure Implications of Secondary Organic Aerosol Production from Indoor Terpene Ozonolysis. *Sci Adv* **2022**, *8* (8), eabj9156.

(27) McDonald, B. C.; De Gouw, J. A.; Gilman, J. B.; Jathar, S. H.; Akherati, A.; Cappa, C. D.; Jimenez, J. L.; Lee-Taylor, J.; Hayes, P. L.; McKeen, S. A. Volatile Chemical Products Emerging as Largest Petrochemical Source of Urban Organic Emissions. *Science (1979)* **2018**, *359* (6377), 760–764.

(28) Gkatzelis, G. I.; Coggon, M. M.; McDonald, B. C.; Peischl, J.; Aikin, K. C.; Gilman, J. B.; Trainer, M.; Warneke, C. Identifying Volatile Chemical Product Tracer Compounds in US Cities. *Environ Sci Technol* **2020**.

(29) Gkatzelis, G. I.; Coggon, M. M.; McDonald, B. C.; Peischl, J.; Gilman, J. B.; Aikin, K. C.; Robinson, M. A.; Canonaco, F.; Prevot, A. S. H.; Trainer, M. Observations Confirm That Volatile Chemical Products Are a Major Source of Petrochemical Emissions in US Cities. *Environ Sci Technol* **2021**, *55* (8), 4332–4343.

(30) Coggon, M. M.; Gkatzelis, G. I.; McDonald, B. C.; Gilman, J. B.; Schwantes, R. H.; Abuhassan, N.; Aikin, K. C.; Arend, M. F.; Berkoff, T. A.; Brown, S. S. Volatile Chemical Product Emissions Enhance Ozone and Modulate Urban Chemistry. *Proceedings of the National Academy of Sciences* **2021**, *118* (32).

(31) Coggon, M. M.; McDonald, B. C.; Vlasenko, A.; Veres, P. R.; Bernard, F.; Koss, A. R.; Yuan, B.; Gilman, J. B.; Peischl, J.; Aikin, K. C. Diurnal Variability and Emission Pattern of Decamethylcyclopentasiloxane (D5) from the Application of Personal Care Products in Two North American Cities. *Environ Sci Technol* **2018**, *52* (10), 5610–5618.

(32) Yang, S.; Muthalagu, A.; Serrano, V. G.; Licina, D. Human Personal Air Pollution Clouds in a Naturally Ventilated Office during the COVID-19 Pandemic. *Build Environ* **2023**, *236*, 110280. https://doi.org/https://doi.org/10.1016/j.buildenv.2023.110280.

(33) Arata, C.; Zarzana, K. J.; Misztal, P. K.; Liu, Y.; Brown, S. S.; Nazaroff, W. W.; Goldstein, A. H. Measurement of NO3 and N2O5 in a Residential Kitchen. *Environ Sci Technol Lett* **2018**, *5* (10), 595–599.

(34) Zannoni, N.; Lakey, P. S. J.; Won, Y.; Shiraiwa, M.; Rim, D.; Weschler, C. J.; Wang, N.; Ernle, L.; Li, M.; Bekö, G. The Human Oxidation Field. *Science (1979)* **2022**, *377* (6610), 1071–1077.

(35) Shiraiwa, M.; Ueda, K.; Pozzer, A.; Lammel, G.; Kampf, C. J.; Fushimi, A.; Enami, S.; Arangio, A. M.; Fröhlich-Nowoisky, J.; Fujitani, Y.; Furuyama, A.; Lakey, P. S. J.; Lelieveld, J.; Lucas, K.; Morino, Y.; Pöschl, U.; Takahama, S.; Takami, A.; Tong, H.; Weber, B.; Yoshino, A.; Sato, K. Aerosol Health Effects from Molecular to Global Scales. *Environ Sci Technol* **2017**, acs.est.7b04417. https://doi.org/10.1021/acs.est.7b04417.

(36) Khan, F.; Kwapiszewska, K.; Zhang, Y.; Chen, Y.; Lambe, A. T.; Kołodziejczyk, A.; Jalal, N.; Rudzinski, K.; Martínez-Romero, A.; Fry, R. C. Toxicological Responses of α-Pinene-Derived Secondary Organic Aerosol and Its Molecular Tracers in Human Lung Cell Lines. *Chem Res Toxicol* **2021**, *34* (3), 817–832.

(37) Nazaroff, W. W. Residential Air-change Rates: A Critical Review. *Indoor Air* **2021**, *31* (2), 282–313.

(38) Li, M.; Weschler, C. J.; Beko, G.; Wargocki, P.; Lucic, G.; Williams, J. Human Ammonia Emission Rates under Various Indoor Environmental Conditions. *Environ Sci Technol* **2020**, *54* (9), 5419–5428.

(39) Cox, R. A.; Ammann, M.; Crowley, J. N.; Herrmann, H.; Jenkin, M. E.; McNeill, V. F.; Mellouki, A.; Troe, J.; Wallington, T. J. Evaluated Kinetic and Photochemical Data for Atmospheric Chemistry: Volume VII–Criegee Intermediates. *Atmos Chem Phys* **2020**, *20* (21), 13497–13519.

(40) Bernard, F.; Daële, V.; Mellouki, A.; Sidebottom, H. Studies of the Gas Phase Reactions of Linalool, 6-Methyl-5-Hepten-2-Ol and 3-Methyl-1-Penten-3-Ol with O3 and OH Radicals. *J Phys Chem A* **2012**, *116* (24), 6113–6126.

(41) Ham, J. E.; Proper, S. P.; Wells, J. R. Gas-Phase Chemistry of Citronellol with Ozone and OH Radical: Rate Constants and Products. *Atmos Environ* **2006**, *40* (4), 726–735.

(42) Forester, C. D.; Ham, J. E.; Wells, J. R. β-Ionone Reactions with Ozone and OH Radical: Rate Constants and Gas-Phase Products. *Atmos Environ* **2007**, *41* (38), 8758–8771.

(43) Aschmann, S. M.; Arey, J.; Atkinson, R.; Simonich, S. L. Atmospheric Lifetimes and Fates of Selected Fragrance Materials and Volatile Model Compounds. *Environ Sci Technol* **2001**, *35* (18), 3595–3600.

(44) Dada, L.; Lehtipalo, K.; Kontkanen, J.; Nieminen, T.; Baalbaki, R.; Ahonen, L.; Duplissy, J.; Yan, C.; Chu, B.; Petäjä, T.; Lehtinen, K.; Kerminen, V.-M.; Kulmala, M.; Kangasluoma, J. Formation and Growth of Sub-3-Nm Aerosol Particles in Experimental Chambers. *Nat Protoc* **2020**, *15* (3), 1013–1040. https://doi.org/10.1038/s41596-019-0274-z.

(45) Zhou, Y.; Dada, L.; Liu, Y.; Fu, Y.; Kangasluoma, J.; Chan, T.; Yan, C.; Chu, B.; Daellenbach, K. R.; Bianchi, F. Variation of Size-Segregated Particle Number Concentrations in Wintertime Beijing. *Atmos Chem Phys* **2020**, *20* (2), 1201–1216.

(46) Jayaratne, R.; Pushpawela, B.; He, C.; Li, H.; Gao, J.; Chai, F.; Morawska, L. Observations of Particles at Their Formation Sizes in Beijing, China. *Atmos Chem Phys* **2017**, *17* (14), 8825–8835.

(47) Yao, L.; Garmash, O.; Bianchi, F.; Zheng, J.; Yan, C.; Paasonen, P.; Sipilä, M.; Wang, M.; Wang, X.; Xiao, S. Atmospheric New Particle Formation from Sulfuric Acid and Amines in a Chinese Megacity. *Science (1979)* **2018**, *281* (July), 278–281. https://doi.org/10.1126/science.aao4839.

(48) Xiao, S.; Wang, M. Y.; Yao, L.; Kulmala, M.; Zhou, B.; Yang, X.; Chen, J. M.; Wang, D. F.; Fu, Q. Y.; Worsnop, D. R.; Wang, L. Strong Atmospheric New Particle Formation in Winter in Urban Shanghai, China. *Atmos Chem Phys* **2015**, *15* (4), 1769–1781. https://doi.org/10.5194/acp-15-1769-2015.

(49) Boulon, J.; Sellegri, K.; Venzac, H.; Picard, D.; Weingartner, E.; Wehrle, G.; Collaud Coen, M.; Bütikofer, R.; Flückiger, E.; Baltensperger, U. New Particle Formation and Ultrafine Charged Aerosol Climatology at a High Altitude Site in the Alps (Jungfraujoch, 3580 m Asl, Switzerland). *Atmos Chem Phys* **2010**, *10* (19), 9333–9349.

(50) Manninen, H. E.; Nieminen, T.; Riipinen, I.; Yli-Juuti, T.; Gagné, S.; Asmi, E.; Aalto, P. P.; Petäjä, T.; Kerminen, V.-M.; Kulmala, M. Charged and Total Particle Formation and Growth Rates during EUCAARI 2007 Campaign in Hyytiälä. *Atmos Chem Phys* **2009**, *9* (12), 4077–4089.

(51) Vana, M.; Komsaare, K.; Hõrrak, U.; Mirme, S.; Nieminen, T.; Kontkanen, J.; Manninen, H. E.; Petäjä, T.; Noe, S. M.; Kulmala, M. Characteristics of New-Particle Formation at Three SMEAR Stations. *Boreal Environment Research* **2016**, *21* (3–4), 345.

(52) Rose, C.; Sellegri, K.; Velarde, F.; Moreno, I.; Ramonet, M.; Weinhold, K.; Krejci, R.; Ginot, P.; Andrade, M.; Wiedensohler, A. Frequent Nucleation Events at the High Altitude Station of Chacaltaya (5240 m Asl), Bolivia. *Atmos Environ* **2015**, *102*, 18–29.

(53) Mohr, C.; Thornton, J. A.; Heitto, A.; Lopez-Hilfiker, F. D.; Lutz, A.; Riipinen, I.; Hong, J.; Donahue, N. M.; Hallquist, M.; Petäjä, T. Molecular Identification of Organic Vapors Driving Atmospheric Nanoparticle Growth. *Nat Commun* **2019**, *10* (1), 4442.
